# From caffeine use motives to sleep disturbance: network and structural equation modeling of caffeine use disorder and depression among caffeine consumers

**DOI:** 10.1016/j.abrep.2026.100729

**Published:** 2026-07-29

**Authors:** Shekh Tanjina Islam Dola, Farhana Najnin, Md Bony Amin, Md. Shamim Sikder, Md. Joy Vangi, Jannatun Naeem, Md. Jahurul Islam Rony, Mst. Mushfika Rahman Takia, Md. Kamruzzaman, Abu Sayeed, Md. Shajadul Islam, Saraban Tahura Ether

**Affiliations:** a Department of Post Harvest Technology, Patuakhali Science and Technology University, Dumki, Patuakhali 8660, Bangladesh; b Department of Food Microbiology and Safety, Patuakhali Science and Technology University, Dumki, Patuakhali 8660, Bangladesh; c Department of Environmental Health and Sanitation, Patuakhali Science and Technology University, Dumki, Patuakhali 8660, Bangladesh; d Faculty of Nutrition and Food Science, Patuakhali Science and Technology University, Dumki, Patuakhali 8660, Bangladesh; e Department of Biochemistry and Molecular Biology, Patuakhali Science and Technology University, Dumki, Patuakhali 8660, Bangladesh; f Faculty of Agriculture, Patuakhali Science and Technology University, Dumki, Patuakhali 8660, Bangladesh; g Maternal and Child Health Division (MCHD), International Centre for Diarrhoeal Disease Research Bangladesh (icddr,b), Dhaka 1212, Bangladesh

**Keywords:** Caffeine use disorder, Sleep problems, Depression, Caffeine use motives, MOS-sleep scale

## Abstract

**Background:**

Caffeine consumption is universal among university students and may be linked with mental health and sleep problems. Yet evidence on the patterns of use, underlying motives, and their links with depression and sleep disturbance among Bangladeshi students is limited.

**Objective:**

This study aimed to investigate the prevalence of caffeine consumption, identify associates, and explore the relationship among caffeine use disorder (CUD), motives, depressive symptoms, and sleep problems.

**Method:**

A cross-sectional study using random sampling was conducted among 530 university students [***M***_***Age***_:21.76 (SD:1.79), 56.8% male]. Participants reported caffeine consumption, motives, depressive and sleep problems symptoms. Network analysis was used to assess item-level connections, and structural equation modeling (SEM) tested direct and indirect pathways.

**Results:**

Nearly 72% of students reported consuming caffeine. Consumption was significantly associated with monthly family expenditure, academic year, smoking habit, and having a loan or debt. Network analysis revealed strong connections among CUD symptoms, particularly impaired control, tolerance, withdrawal, and continued use despite harm. Craving and self-reward motives acted as key bridge nodes. Depressive symptoms and sleep problems were closely linked, with clinically relevant bridges involving suicidal ideation and concentration difficulties. SEM showed that CUD had a significant direct effect on sleep problems and an indirect effect through depressive symptoms, while motives did not directly affect sleep disturbance.

**Conclusion:**

Visible clusters among symptoms and CUD's direct and indirect effects through depression on sleep problems highlight the need for psychoeducation on healthy caffeine consumption and screening for mental health problems for better sleep quality.

## Highlights

• High prevalence (72%) of caffeine use among university students.
• Craving and self-reward bridge CUD, depression, and sleep problems.
• CUD directly and indirectly affects sleep via depression.
• Novel evidence from Bangladesh using network and SEM analyses.

## 1 Introduction

Caffeine is a natural stimulant that affects the central nervous system. While it is mainly found in coffee beans, it also occurs naturally in some teas and cacao beans and is added to sodas and energy drinks (Evans, Richards, & Battisti, 2024). Oral caffeine has been licensed by the US Food and Drug Administration (FDA) to help people who are tired or drowsy regain their mental alertness or wakefulness. Additionally, the FDA has authorized intravenous (IV) caffeine for the treatment of preterm apnea (Long et al., 2021; Mathew, 2011; Schmidt, 2005). Many people, especially young adults, use caffeine to increase alertness and reduce tiredness. Among university students, caffeine use is common because of academic demands and long study hours (Bertasi et al., 2021).

In student populations, sleep problems are also common. Poor sleep quality can reduce daily functioning, impair mood, and increase the risk of depression (Whalen et al., 2008). Depression itself is a major concern among students, as it can affect performance, social life, and overall, well-being (Hamdan et al., 2025). Previous studies have shown links between caffeine use, sleep disturbance, and depressive symptoms. For example, higher caffeine consumption has been associated with poorer sleep and greater psychological distress in student samples (Bertasi et al., 2021). However, most past research has focused on the amount of caffeine consumed or on simple associations with sleep and mood. Few studies have examined why students consume caffeine, how their motives relate to psychological outcomes, or how different symptoms interconnect. Motivations for caffeine use, such as alertness enhancement, mood regulation, social reasons, weight control, or withdrawal relief, can influence consumption patterns and their effects on mental health (Choi, 2020; Nova, Hernandez, Ptolemy, & Zeitzer, 2012). Understanding these motives can help identify high-risk groups and inform targeted strategies for healthier consumption.

Caffeine use disorder (CUD) is a condition in which individuals have difficulty controlling their caffeine intake despite negative effects. CUD has been proposed in diagnostic manuals but remains under-studied, especially in young adults (Sweeney, Weaver, Vincent, Arria, & Griffiths, 2020). CUD symptoms have been linked with greater emotional distress and poorer sleep in general population samples (AlSaleh et al., 2025; Sweeney et al., 2020). For instance, the development and validation study of caffeine use disorder and caffeine addiction scale found a significant correlation with anxiety, highlighting the importance of considering problematic use patterns when studying caffeine and mental health (AlSaleh et al., 2025).

Evidence from prior studies suggests that constructs such as caffeine use motives, CUD symptoms, mental health, and sleep problems are interlinked; however, they have not been examined in a single framework (AlSaleh et al., 2025; Sweeney et al., 2020). Additionally, previous research has focused on these factors in isolation without considering their interactions at the item level (Sweeney et al., 2020). Therefore, there is a considerable ‘gap in knowledge’ concerning which particular symptoms function as the key mediators between emotional instability and excessive caffeine intake. This is particularly true for low- and middle-income countries, including Bangladesh, where this topic is under-studied and where student lifestyle and academic stress may not be comparable to those of the West.

By integrating these constructs within a single, theoretically sound model, this study aims to take its investigation further than just establishing correlations. In this way, we will investigate the relationships among caffeine use motives, caffeine use disorders (CUD), depression, and sleep problems using sophisticated network analysis and structural equation modeling (SEM). Network analysis will assist in exploring interrelationships among these symptoms and behaviors, while SEM will assist in exploring direct and indirect effects of these item constructs. Additionally, we will examine the prevalence and associates of caffeine consumption using the complete dataset. Together, these analyses will enable us to gain deeper knowledge than simple correlation analysis.

## 2 Method

### 2.1 Study design and participants

We conducted a cross-sectional survey among university students in the Barishal division of Bangladesh from November 10, 2025, to January 24, 2026. The Barishal division has two public universities: Patuakhali Science and Technology University and University of Barisal. We included enrolled students from both universities. The first objective was to estimate the prevalence of caffeine consumption and identify its associated factors. The second objective was to examine the network structure of caffeine use motives, caffeine use disorder symptoms, depression, and sleep problems. The final objective was to assess the direct and indirect effects of caffeine use motives, caffeine use disorder symptoms, and depression on sleep problems. We excluded individuals with pre-existing disabilities that affected mobility, speech, hearing, or vision to minimize potential confounding and measurement bias, as these conditions may independently influence mental health and sleep outcomes and could also affect the accurate completion of the study. We also excluded students who were taking painkillers or caffeine-containing medications, such as paracetamol, at the time of the survey.

### 2.2 Sampling and settings

We used probability sampling (simple random sampling) to collect the data. The required sample size was calculated using the standard formula for cross-sectional studies with a 5% margin of error. Assuming a 50% prevalence of caffeine consumption and a 95% confidence level, the minimum required sample size was 385 (Bartlett, Kotrlik, & Higgins, 2001). We collected data in classrooms after lectures. We used the attendance sheets as the sampling frame. From each class, we selected 50% of the students using a lottery method to ensure that every student had an equal chance of selection. We then invited the selected students to participate and obtained informed consent before data collection. If a selected student declined, we did not include that individual. We continued this process until we obtained 550 responses. After data cleaning and screening, 530 responses remained and were included in the final analysis.

### 2.3 Ethical approval

This study involved human participants. We did not conduct any experiments or provide any interventions. We informed all participants about the study before data collection. We obtained both oral and written informed consent from each participant. We also obtained permission from the university authorities to go to the classrooms. The authors assert that all procedures contributing to this work comply with the ethical standards of the relevant national and institutional committees on human experimentation and with the Helsinki Declaration of 1975, as revised in 2013. All procedures involving human subjects/patients were approved by Patuakhali Science and Technology University **(approval no.** **PSTU/IEC/2025/44(ii)).**

### 2.4 Questionnaire

We developed and administered a structured questionnaire. It had five sections. The first section included socio-demographic information and general questions on caffeine use (e.g., “Do you consume caffeine?” and frequency of consumption in cups, equivalent to 80–100 mL small cups). If a participant reported caffeine use, they completed the remaining sections. We assessed caffeine use disorder using the Caffeine Use Disorder Questionnaire (CUDQ), caffeine intake motives using the Caffeine Motives Questionnaire (CMQ), sleep problems using the MOS Sleep Scale (MOSS), and depressive symptoms using the Patient Health Questionnaire-9 (PHQ-9). The scale items are presented in **Supplementary Tables S1–S4**. Because the MOS Sleep, CUDQ, and CMQ scales were new to this setting, we sought expert opinion to ensure item relevance before finalizing the questionnaire. We first prepared the questionnaire in English. We then translated it into Bangla and back-translated it into English. One subject expert and one professional translator reviewed and revised the translation. We conducted a pilot test with 25 participants to assess reliability. We calculated internal consistency using Cronbach's alpha. We excluded pilot participants from the final analysis. We coded all scale items and provided them in the supplementary tables.

#### 2.4.1 Sociodemographic and other variables

We collected data on age, gender, academic year, marital status, current residence, and permanent residence. We also recorded financial information, including monthly average family income, monthly average family expenditure, and current loan or debt status. Academic and work-related information included cumulative grade point average (CGPA) and part-time job status. We gathered lifestyle and health-related data. These included smoking status, alcohol use, presence of non-communicable diseases (NCDs), current medication use, family history of sleep disorders, and physical activity. We defined adequate physical activity as at least 150 min of exercise in the past week. We treated age, monthly family income, monthly family expenditure, and CGPA as continuous variables. We collected all other socio-demographic and lifestyle variables as categorical variables.

#### 2.4.2 CMQ-21

The Caffeine Motives Questionnaire-21 (CMQ-21) is a self-report instrument designed to assess the underlying motives for caffeine consumption. The scale consists of 21 items that evaluate different motivational dimensions, including alertness enhancement, mood regulation, social motives, weight control, and withdrawal relief. However, in the present study, we used this scale to examine the network structure and to derive a single latent factor. Participants rate each item on a 5-point Likert scale ranging from 1 (strongly disagree) to 5 (strongly agree), with higher scores indicating stronger endorsement of caffeine use motives (Irons et al., 2014). The internal consistency of the CMQ-21 was 0.915.

#### 2.4.3 CUDQ-10

The Caffeine Use Disorder Questionnaire-10 (CUDQ-10) is a self-reported scale developed to assess symptoms of caffeine use disorder. The scale consists of 10 items measuring features such as impaired control over caffeine use, tolerance, withdrawal, continued use despite harm, and functional impairment. Participants responded to each item using a 4-point Likert scale ranging from 0 (Never) to 3 (Always), with higher scores indicating greater severity of caffeine-related problems (Ágoston, Urbán, Richman, & Demetrovics, 2018). In the present study, the internal consistency of the CUDQ-10 demonstrated acceptable reliability, with a Cronbach's alpha of 0.764.

#### 2.4.4 MOS-sleep scale

The Medical Outcomes Study (MOS) Sleep Scale is a self-reported instrument designed to assess multiple aspects of sleep over the past four weeks. Participants responded to items on various scales (e.g., minutes, hours, or a 6 point Likert scale), with a scoring process that involves two steps: first, certain items (e.g., MOSS_03, MOSS_07, MOSS_10) are reverse scored so that, generally, a higher final score reflects more sleep problems or a greater severity of the attribute; second, all item scores are standardized to a 0 to 100 range. The final scale score is the average of the standardized scores for the items within that specific dimension, where a score of 100 represents the highest possible level of the problem. MOSS_02 was excluded from the scale and used only for measuring sleeping adequacy (7 to 8 h sleep) (Shahid, Wilkinson, Marcu, & Shapiro, 2011). This scale's Cronbach's alpha (11 items) was 0.728.

#### 2.4.5 PHQ-9

The Patient Health Questionnaire-9 is a widely used self-report measure of depressive symptoms over the past two weeks. It includes nine items based on the diagnostic criteria for major depressive disorder. Participants rated each item on a 4-point Likert scale from 0 (not at all) to 3 (nearly every day). Higher scores indicate greater symptom severity (Kroenke, Spitzer, & Williams, 2001; Rahman, Dhira, Sarker, & Mehareen, 2022). In this study, the PHQ-9 showed good internal consistency (Cronbach's α = 0.857).

### 2.5 Statistical analyses

We performed all statistical analyses using R version 4.4.0 (via RStudio) and SPSS version 27.0. We first cleaned the data by identifying and removing records with missing values and by screening the dataset to ensure completeness and quality of the final analytical sample. We then computed descriptive statistics (frequencies, percentages, means, and standard deviations). Next, we examined bivariate associations between caffeine intake and other variables using the chi-square (χ^2^) test. We then performed unadjusted and adjusted logistic regression analyses to identify associates of caffeine consumption. Variables with *p* values less than 0.250 in the unadjusted model were included in the multivariable model to control for potential confounding effects. We checked the assumptions of logistic regression, including multicollinearity and model fit. The final model showed acceptable fit (Hosmer–Lemeshow test: χ^2^ = 6.49, df = 8, *p* = 0.592).

For network analysis and structural modeling (*n* = 383), we excluded non-caffeine consumers to ensure meaningful estimation and pathway relationships among caffeine-related constructs. We estimated a symptom-level network using “non-parametric correlations”. Then, we carried out the centrality indices and bridge metrics to identify the most influential and connecting nodes in the network. We then conducted structural equation modeling (SEM) to examine the direct and indirect effects of caffeine use disorder (CUDQ), caffeine-intake motives (CMQ), and depressive symptoms (PHQ-9) on sleep problems (MOS Sleep). We evaluated model fit using multiple indices. The model showed mixed fit: χ^2^ = 3625.17, CFI = 0.683, TLI = 0.668, RMSEA = 0.07, and SRMR = 0.08 (Amin et al., 2026). The packages, along with all codes, settings, and outputs, are provided in the supplementary materials (See supplementary table S5). We conducted network analysis, SEM, and visualization using R packages such as haven (v2.5.4), dplyr (v1.1.4), psych (v2.4.3), qgraph (v1.9.8), networktools (v1.5.2), and lavaan (v0.6.18) (Anderson & Gerbing, 1988; Briganti et al., 2024). All tests were two-tailed, and we considered *p* < 0.05 as statistically significant.

## 3 Result

Table 1 describes the sociodemographic characteristics and their caffeine consumption status. The mean age of the participants was 21.76 (SD:1.79). Among 530 participants, 72.3% reported caffeine consumption. Most were aged 21–24 years (64.5%) and male (56.8%). Caffeine consumers reported to ingest an average of 1.9 (SD:1.11) cups of caffeine drinks (tea/coffee). Caffeine intake was higher among smokers (85.4%) than among non-smokers (71.0%), and among those with loans or debt (82.7%) than among those without (69.9%). Smoking (χ^2^ = 4.56, *p* = 0.033) and having a loan or debt (χ^2^ = 6.47, *p* = 0.011) were significantly associated with caffeine consumption. No significant associations were observed for age, gender, residency, income, expenditure, academic year, marital status, sleep, alcohol use, NCDs, medication use, family history of sleep disorder, or physical exercise (*p* > 0.05).

**Table 1:** Information about sociodemographic and caffeine consumption based on the total sample (*n* = 530).

Variables	Categories	Total (%)	Consumption of caffeine (%)	χ^2^	*p*-value
Age in years *M*_age_ = 21.76 (SD: 1.79)	18 to 20	140 (26.4)	99 (70.7)	3.25	0.197
	21 to 24	342 (64.5)	244 (71.3)		
	25 to 30	48 (9.1)	40 (83.3)		
Gender	Male	301 (56.8)	224 (74.4)	1.61	0.204
	Female	229 (43.2)	159 (69.4)		
Current residency	University hall	328 (61.9)	229 (69.8)	3.89	0.274
	Rental house with friends	138 (26)	106 (76.8)		
	Rental house alone	32 (6)	22 (68.8)		
	House with family	32 (6)	26 (81.3)		
Permanent residency	Rural area	339 (64)	244 (72)	0.04	0.844
	Urban area	191 (36)	139 (72.8)		
Monthly average family income	Below 15,000 BDT	67 (12.6)	53 (79.1)	1.87	0.393
	15,000 to 30,000 BDT	290 (54.7)	208 (71.7)		
	Above 30,000 BDT	173 (32.6)	122 (70.5)		
Monthly average family expenditure	Below 15,000 BDT	100 (18.9)	81 (81)	5.08	0.079
	15,000 to 30,000 BDT	311 (58.7)	221 (71.1)		
	Above 30,000 BDT	119 (22.5)	81 (68.1)		
Academic year	First	246 (46.4)	175 (71.1)	5.43	0.246
	Second	104 (19.6)	74 (71.2)		
	Third	66 (12.5)	43 (65.2)		
	Fourth	63 (11.9)	49 (77.8)		
	Master's	51 (9.6)	42 (82.4)		
Marital status	Unmarried	472 (89.1)	341 (72.2)	0.02	0.992
	Married	54 (10.2)	39 (72.2)		
	Divorced/Widowed	4 (0.8)	3 (75)		
Have any part-time job	No	376 (70.9)	268 (71.3)	0.63	0.427
	Yes	154 (29.1)	115 (74.7)		
CGPA above 3.00	No	40 (7.5)	32 (80)	1.29	0.256
	Yes	490 (92.5)	351 (71.6)		
Inadequate sleep	No	181 (34.2)	137 (75.7)	1.61	0.204
	Yes	349 (65.8)	246 (70.5)		
Habit of smoking	No	482 (90.9)	342 (71)	4.56	**0.033**
	Yes	48 (9.1)	41 (85.4)		
Alcohol consumption	No	504 (95.1)	364 (72.2)	0.009	0.924
	Yes	26 (4.9)	19 (73.1)		
Have a loan/debt	No	432 (81.5)	302 (69.9)	6.47	**0.011**
	Yes	98 (18.5)	81 (82.7)		
Have NCDs	No	454 (85.7)	328 (72.2)	0.01	0.982
	Yes	76 (14.3)	55 (72.4)		
Currently under medication	No	428 (80.8)	307 (71.7)	0.32	0.573
	Yes	102 (19.2)	76 (74.5)		
Family history of a sleeping disorder	No	354 (66.8)	250 (70.6)	1.44	0.231
	Yes	176 (33.2)	133 (75.6)		
Minimum 150 min of physical exercise in the past week	No	332 (62.6)	238 (71.7)	0.15	0.701
	Yes	198 (37.4)	145 (73.2)		
Average frequency of caffeine consumption (equivalent to 80–100 ml small cups); *n* = 382, missing = 1			Mean ± SD: 1.9 ± 1.11		
Total		530 (100)	383 (72.3)		

Note: χ^2^ refers to the Chi-square test statistic, and *M*_age_ refers to mean age.

Table 2 presents the results of logistic regression analyses identifying the associates of caffeine consumption. In the unadjusted model, participants with lower monthly family expenditure (<15,000 BDT) had a 2-fold higher likelihood of caffeine consumption compared with those with higher expenditure (>30,000 BDT) (OR = 2.00, 95% CI: 1.06–3.76, *p* = 0.031). This association remained significant after adjustment, where participants with lower monthly family expenditure had a 2.5-fold higher likelihood of caffeine consumption (AOR = 2.65, 95% CI: 1.01–6.92, *p* = 0.047). Non-smokers had a 58% lower chance of caffeine consumption than smokers in the crude model (OR = 0.42, 95% CI: 0.18–0.95, *p* = 0.038), but lost significance after adjustment. Participants without any loans or debts had a51% and 48% lower chance of caffeine intake in the crude (OR = 0.49, 95% CI: 0.28–0.86, *p* = 0.012) and adjusted models (AOR = 0.52, 95% CI: 0.29–0.95, *p* = 0.032), respectively.

**Table 2:** Associates of caffeine consumption based on the total sample (n = 530).

		Caffeine Consumption^a^			
Variables	Categories	*p-value*	OR 95% CI [LL - UL]	*p-value*	AOR 95% CI [LL - UL]
Age in years *M*_age_ = 21.76 (SD: 1.79)	18 to 20	0.090	0.48 [0.21–1.12]	0.522	0.67 [0.20–2.27]
	21 to 24	0.085	0.50 [0.23–1.1]	0.522	0.69 [0.22–2.15]
	25 to 30				
Gender	Male	0.204	1.28 [0.87–1.88]	0.791	0.94 [0.59–1.49]
	Female				
Current residency	University hall	0.180	0.53 [0.21–1.34]	0.367	0.64 [0.25–1.68]
	Rental house with friends	0.588	0.76 [0.29–2.02]	0.914	0.95 [0.34–2.62]
	Rental house alone	0.252	0.51 [0.16–1.62]	0.390	0.59 [0.18–1.96]
	House with family				
Permanent residency	Rural area	0.844	0.96 [0.65–1.43]		
	Urban area				
Monthly average family income	Below 15,000 BDT	0.182	1.58 [0.81–3.1]	0.538	0.73 [0.26–2.0]
	15,000 to 30,000 BDT	0.782	1.06 [0.7–1.61]	0.580	0.84 [0.46–1.55]
	Above 30,000 BDT				
Monthly average family expenditure	Below 15,000 BDT	**0.031**	**2.0 [1.06–3.76]**	**0.047**	**2.65 [1.01–6.92]**
	15,000 to 30,000 BDT	0.544	1.15 [0.73–1.82]	0.325	1.39 [0.72–2.65]
	Above 30,000 BDT				
Academic year	First	0.105	0.53 [0.24–1.14]	0.787	0.85 [0.26–2.76]
	Second	0.135	0.53 [0.23–1.22]	0.714	0.80 [0.25–2.62]
	Third	**0.042**	**0.4 [0.17–0.97]**	0.592	0.71 [0.20–2.50]
	Fourth	0.546	0.75 [0.30–1.91]	0.790	1.18 [0.35–4.02]
	Master's				
Marital status	Unmarried	0.903	0.87 [0.09–8.42]		
	Married	0.905	0.87 [0.08–9.0]		
	Divorced/Widowed				
Have any part-time job	No	0.428	0.84 [0.55–1.29]		
	Yes				
CGPA above 3.00	No	0.250	1.58 [0.71–3.52]	0.441	1.39 [0.61–3.17]
	Yes				
Inadequate sleep	No	0.205	1.3 [0.87–1.97]	0.417	1.2 [0.77–1.88]
	Yes				
Habit of smoking	No	**0.038**	**0.42 [0.18–0.95]**	0.140	0.5 [0.2–1.25]
	Yes				
Alcohol consumption	No	0.924	0.96 [0.39–2.33]		
	Yes				
Have a loan/debt	No	**0.012**	**0.49 [0.28–0.86]**	**0.032**	**0.52 [0.29–0.95]**
	Yes				
Have NCDs	No	0.982	0.99 [0.58–1.71]		
	Yes				
Currently under medication	No	0.573	0.87 [0.53–1.42]		
	Yes				
Family history of a sleeping disorder	No	0.232	0.78 [0.51–1.18]	0.409	0.83 [0.53–1.29]
	Yes				
Minimum 150 min of physical exercise in the past week	No	0.701	0.93 [0.62–1.37]		
	Yes				

**Note:** Variables with a p-value <0.25 in the unadjusted model were included in the adjusted model to address potential confounding effects. OR = Odds Ratio; AOR = Adjusted Odds Ratio; CI = Confidence Interval; LL = Lower Limit; UL = Upper Limit.

a The reference category is: No.

**Fig. 1:**
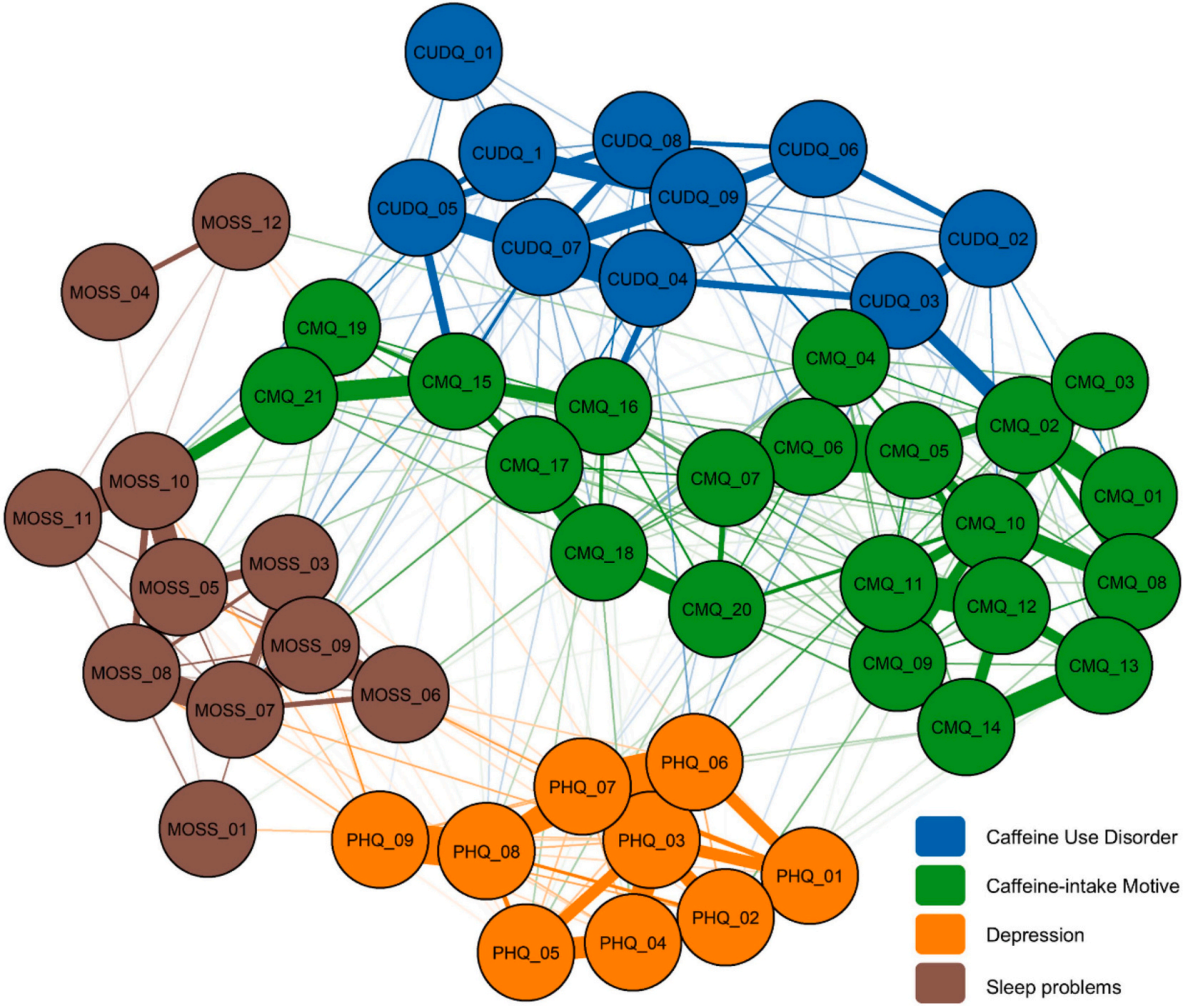
Network structure of caffeine addiction, motive of intake, sleep problems, and depression based on caffeine users (*n* = 383).**Note:** CUDQ: Caffeine Use Disorder Questionnaire; CMQ: Caffeine-intake Motive Questionnaire; MOSS: Medical Outcome Study Sleep; PHQ: Depression.

Fig. 1 presents the network structure among caffeine use disorder, caffeine-intake motives, sleep problems, and depression items among caffeine consumers. Nodes represent individual items, and edges represent regularized partial correlations. Thicker edges indicate stronger associations. Within the caffeine use disorder cluster, strong connections were observed, particularly among CUDQ_04, CUDQ_05, CUDQ_07, CUDQ_08, and CUDQ_09, showing close interrelations between impaired control, tolerance, withdrawal, social/interpersonal problems, and continued use despite harm. Within the motive cluster, strong associations were found among CMQ_05, CMQ_06, CMQ_07, and CMQ_10, showing focus and mood-related motives (depression, anxiety, and stress management). CMQ_11 (motive for energy), CMQ_12 (relax and calm), and CMQ_13 (staying awake) formed another cohesive group, while CMQ_16, CMQ_17, and CMQ_18 also showed dense interconnections, depicting craving and self-rewarding for completing a task. In the depression cluster, strong edges were observed between PHQ_01, PHQ_02, PHQ_03, PHQ_04, PHQ_05, and PHQ_07, core depressive symptoms, including low mood, anhedonia, sleep disturbance, fatigue, appetite changes, and concentration problems. Within the sleep problem cluster, strong associations were seen among MOSS_05, MOSS_06, MOSS_07, MOSS_08, MOSS_09, and MOSS_10, representing sleep disturbance and sleep adequacy components. Bridge connections were prominently observed between CUDQ and CMQ items, particularly impaired controls (CUDQ_04 and CUDQ_05), showing notable cross-links with craving and self-rewarding for completing a task (CMQ_17 and CMQ_16). Furthermore, PHQ_09 (suicidal ideation) and PHQ_07 (concentration difficulties) related to MOSS_06 (daytime fatigue) and MOSS_07 (trouble falling asleep), showing overlap between depressive symptoms and sleep problems.

**Fig. 2:**
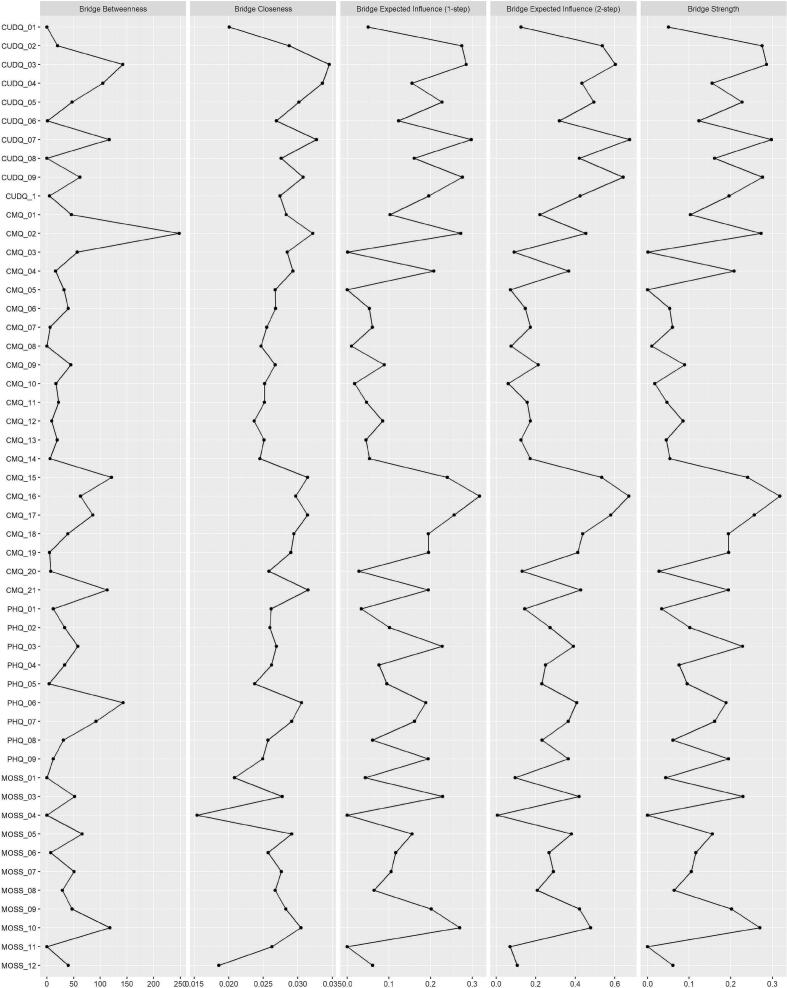
Centrality indices of the network structure based on caffeine users (n = 383).**Note:** CUDQ: Caffeine Use Disorder Questionnaire; CMQ: Caffeine-intake Motive Questionnaire; MOSS: Medical Outcome Study Sleep; PHQ: Depression. There are no predefined threshold values for these indices, and presented for comparative visualization across items.

Fig. 2 shows the bridge centrality indices, including bridge betweenness, bridge closeness, bridge expected influence (1-step), bridge expected influence (2-step), and bridge strength. There is no available predefined threshold for these indices. Bridge centrality indices quantify the extent to which a node connects different symptom communities, with higher values indicating greater bridging potential between constructs. CMQ_16 (because I crave caffeine) showed the highest bridge strength (0.318). This was followed by CUDQ_07 (tolerance; 0.298), CUDQ_03 (use to avoid withdrawal; 0.286), and CUDQ_09 (strong desire or urge; 0.276). CUDQ_02 (continued use despite harm; 0.275) and CMQ_02 (to combat a headache; 0.273) also showed high bridge values. Among sleep items, MOSS_10 (sleep disturbance; 0.270) and MOSS_03 (sleep adequacy; 0.229) were prominent. CMQ_17 (as a reward; 0.256) and CMQ_15 (ingredient in diet pills; 0.240) also demonstrated notable bridge strength.

**Fig. 3:**
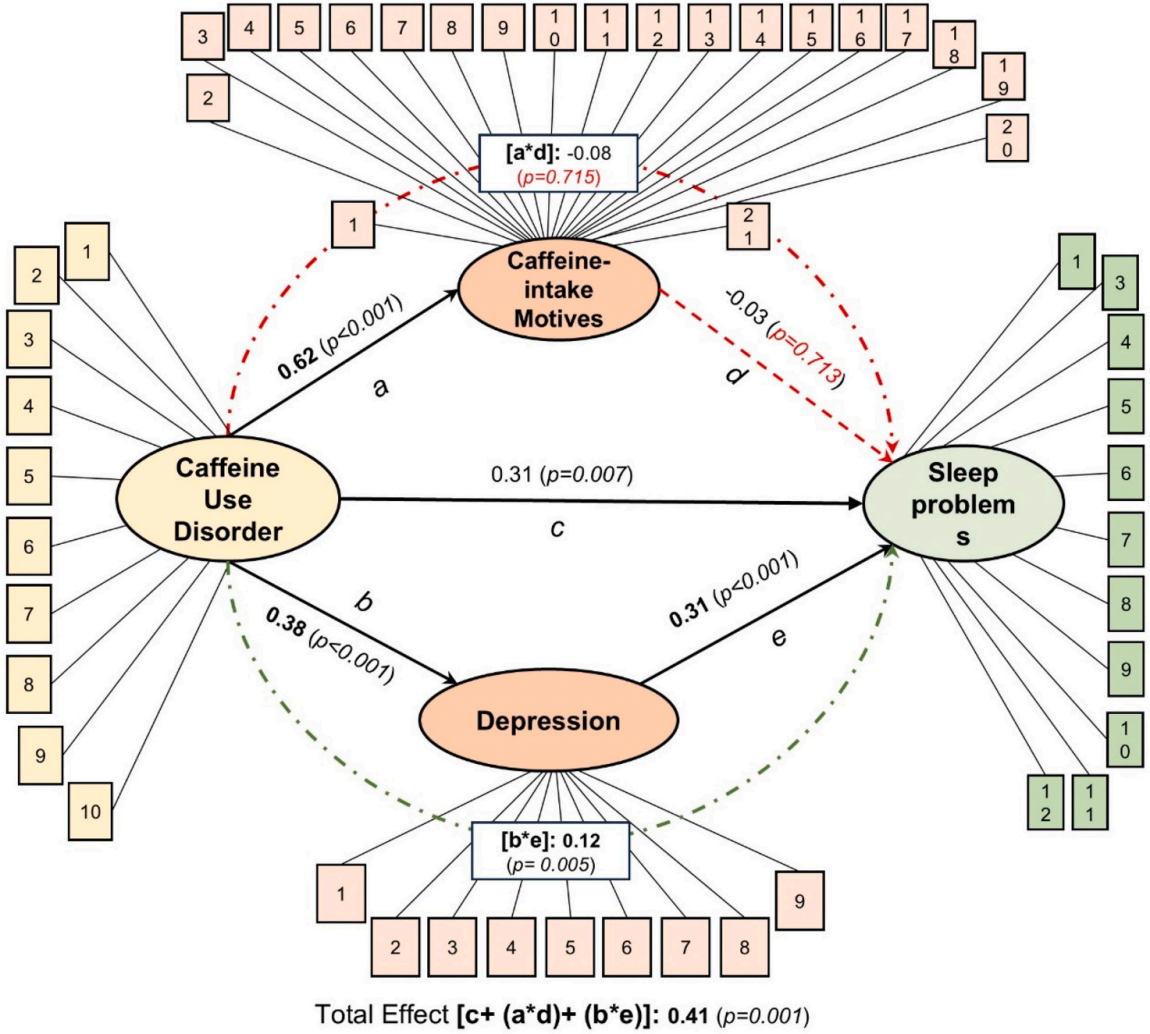
Direct and indirect effects of Caffeine Use Disorder, Motives, and Depression, on Sleep problems based on caffeine users (n = 383).**Note:** CUDQ = Caffeine Use Disorder; CMQ = Caffeine-intake Motive; MOSS = Medical Outcome Study Sleep (Sleep problems); PHQ = Depression. Direct effects are shown by single letters, whereas indirect effects are represented by combinations of letters. For example, c denotes the direct effect of CUD on sleep problems, while b*e represents the indirect effect of CUD on sleep problems through depression.

Fig. 3 reveals the structural equation model. An example of interpretation is that a one standard deviation increase in the latent construct leads to a change in the outcome measured in standard deviation units (β). If the β is positive, the outcome increases; if β is negative, the outcome decreases. Caffeine use disorder had a significant positive effect on caffeine-intaking motives (***β*** = 0.62, *p* < 0.001) and depression (***β*** = 0.38, p < 0.001). Specifically, a one standard deviation increase in caffeine use disorder was associated with a 0.62 standard deviation increase in caffeine-intaking motives and a 0.38 standard deviation increase in depression scores. Caffeine use disorder also demonstrated a significant direct effect on sleep problems (MOS) (***β*** = 0.31, *p* = 0.007). Caffeine-intaking motives did not significantly predict MOS (β = −0.03, *p* = 0.713). However, depression showed a significant positive effect on sleep problems (***β*** = 0.31, p < 0.001). Regarding mediation effects, the indirect effect of caffeine use disorder on sleep problems through motives was not significant (***β*** = −0.02, *p* = 0.715). In contrast, the indirect effect through depression was significant (***β*** = 0.12, *p* = 0.005). The total effect of caffeine use disorder on sleep problems was significant (***β*** = 0.41, *p* = 0.001).

## 4 Discussion

Our study explored the prevalence of caffeine consumption and its associated factors among university students in Bangladesh. Nearly three-quarters of the students reported consuming caffeine. This prevalence is comparable to findings from studies conducted in university settings in different countries (Mackus, van de Loo, Benson, Scholey, & Verster, 2016; Mahoney et al., 2019; Riera-Sampol, Rodas, Martínez, Moir, & Tauler, 2022). In addition, this study examined relationships at the item level, such as specific motives and symptoms, using network analysis. We also tested direct and indirect effects among motives, caffeine use disorder (CUD) symptoms, depression, and sleep problems using structural equation modeling (SEM).

Our findings showed no significant association between caffeine consumption and most sociodemographic variables. However, monthly family expenditure, academic year, smoking habit, and having a loan or debt were significantly associated with caffeine use. Financial difficulties, academic pressure, and debt may increase psychological stress, which may lead to higher caffeine intake. Previous studies have also linked caffeine consumption with depressive symptoms and stress among university students (Arslan & Aydemir, 2021; Nadeem, Khan, Tanweer, Dar, & Tariq, 2025). Riera-Sampol et al. (2022) reported that smoking is more likely to be associated with caffeine consumption (Riera-Sampol et al., 2022). In contrast, age, gender, and CGPA were not significantly associated with caffeine use in our study. These findings are consistent with previous studies (Arslan & Aydemir, 2021; Nadeem et al., 2025). In this population, caffeine use may be a common habit. Cultural normalization may also make caffeine consumption a socially accepted behavior. Our findings regarding age differ from those reported by Riera-Sampol et al. (2022) (Riera-Sampol et al., 2022). This difference may be due to the narrow age range in our sample and the university-based setting. Future studies should include wider age groups and different occupational groups to better understand these patterns.

Network analysis showed strong connections within the CUD symptom cluster. Impaired control, tolerance, withdrawal, and continued use despite harm were closely linked. Similar clustering of CUD and psychiatric symptoms has been reported in recent research (Davoudi, Abdoli, Momeni, & Asgarabad, 2025). Within the motive cluster, craving and self-reward motives showed high bridge centrality. Craving and withdrawal-related use were also important bridge nodes. These findings are consistent with prior evidence highlighting their role in problematic caffeine use (Davoudi et al., 2025). Such patterns are similar to those observed in other substance use disorders. In addition, these results suggest that caffeine use disorder may be maintained through mutually reinforcing symptom interactions, where closely connected symptoms strengthen each other over time. The identification of highly central and bridging nodes indicates potential targets for intervention, particularly focusing on craving regulation and withdrawal management. This is necessary, according to the network theory of psychology, to disrupt the cross-cluster symptom severity and activation (Borsboom, 2017).

Depressive symptoms and sleep problems were strongly interconnected. Strong links between core depressive symptoms and sleep disturbance are consistent with previous findings among university students (Akova et al., 2023; Isa et al., 2021). Many studies have shown that caffeine consumption is associated with poor sleep quality and higher levels of depressive symptoms (Riera-Sampol et al., 2022; Whalen et al., 2008). The bridge connections between suicidal ideation, concentration problems, and sleep disturbance are clinically important. Overlapping sleep and mood symptoms may increase psychological vulnerability. SEM further clarified these relationships. CUD had a significant direct effect on sleep problems and an indirect effect through depressive symptoms. In contrast, motives did not directly predict sleep disturbance. Previous research suggests that caffeine intake may impair sleep quality, which may then worsen depressive symptoms (Kim et al., 2025; Riera-Sampol et al., 2022). Our findings suggest that depressive symptoms may partially mediate the relationship between problematic caffeine use and sleep problems. These findings have important practical implications. Universities should provide psychoeducation on healthy caffeine use and screen for depressive symptoms among students who are at high risk.

This study has several limitations. First, the cross-sectional design does not allow us to draw causal conclusions. Second, all data were self-reported, which may introduce recall and social desirability bias. Third, the sample was taken from only one division of Bangladesh, which may limit the generalizability of the findings to other regions. Longitudinal studies are needed to examine temporal relationships and to test the effects of possible interventions. Caffeine intake was not differentiated by specific sources such as tea, coffee, energy drinks, or caffeinated soft drinks. Given that caffeine content varies considerably across beverages and consumption patterns may differ among young adults, the lack of source-specific assessment may have introduced variability in estimates of actual caffeine exposure. We were also unable to measure the exact amount of caffeine intake because there is no validated caffeine use scale in Bangladesh, and there is no standard caffeine content in locally prepared coffee. In addition, the SEM model showed CFI and TLI values that were slightly below the recommended thresholds, which may limit the strength of some pathways and node connections. Despite these limitations, this study combines network analysis and SEM within a single analytical framework. The findings suggest that depressive symptoms play a central role in linking caffeine use disorder and sleep problems and may help inform prevention strategies and student mental health programs.

## 5 Conclusion

This study found a high prevalence of caffeine consumption among university students in Bangladesh and identified important psychological correlates, particularly depression and sleep problems. Network analysis suggests that certain features, especially craving and withdrawal-related symptoms, may play a key role in connecting caffeine use disorder with mental health and sleep-related outcomes. Furthermore, the findings highlight the role of depressive symptoms in linking problematic caffeine use and poor sleep. Overall, these results underscore the importance of addressing caffeine-related behaviors within student populations, alongside routine mental health screening. Universities should promote awareness of healthy caffeine habits and integrate mental health screening to reduce sleep disturbances and psychological distress among students.

## CRediT authorship contribution statement

**Shekh Tanjina Islam Dola:** Writing – review & editing, Writing – original draft, Visualization, Methodology, Data curation, Conceptualization. **Farhana Najnin:** Writing – review & editing, Writing – original draft, Visualization, Methodology, Data curation, Conceptualization. **Md Bony Amin:** Writing – review & editing, Writing – original draft, Visualization, Validation, Methodology, Formal analysis, Data curation, Conceptualization. **Md. Shamim Sikder:** Writing – review & editing, Investigation, Data curation. **Md. Joy Vangi:** Writing – review & editing, Investigation, Data curation. **Jannatun Naeem:** Writing – review & editing, Investigation, Data curation. **Md. Jahurul Islam Rony:** Writing – review & editing, Investigation, Data curation. **Mst. Mushfika Rahman Takia:** Writing – review & editing, Investigation, Data curation. **Md. Kamruzzaman:** Validation, Writing – review & editing. **Abu Sayeed:** Writing – review & editing, Validation. **Md. Shajadul Islam:** Writing – review & editing, Validation. **Saraban Tahura Ether:** Validation, Writing – review & editing.

## Declaration of generative AI and AI-assisted technologies in the writing process

During the preparation of this work, the authors used ChatGPT to improve the wording. After using this tool, the authors reviewed and edited the content as needed and took full responsibility for the publication's content.

## Funding

The authors did not receive any internal or external funds.

## Declaration of competing interest

The authors declare that they have no known competing financial interests or personal relationships that could have appeared to influence the work reported in this paper.

## Data Availability

Data will be made available on request.
