# Supplementary material for: From caffeine use motives to sleep disturbance: network and structural equation modeling of caffeine use disorder and depression among caffeine consumers
Source: Addict Behav Rep. 2026 Jul 29;24:100729. doi: 10.1016/j.abrep.2026.100729 (PMC13452143; doi:10.1016/j.abrep.2026.100729)
Supplement: Supplementary file 1 [file mmc1.pdf]

From Caffeine Use Motives to Sleep Disturbance: Network and Structural Equation Modeling of Caffeine Use Disorder and Depression Among Caffeine Consumers

Appendix

Table S1. Items of CMQ-21

**I choose to ingest caffeine….**
**CMQ_01:** To feel more alert
**CMQ_02:** To combat a headache
**CMQ_03:** To help me concentrate
**CMQ_04:** Because I like the taste of caffeinated beverages
**CMQ_05:** To help deal with stress in my daily life
**CMQ_06:** To help deal with anxiety
**CMQ_07:** To help deal with depression
**CMQ_08:** To combat drowsiness
**CMQ_09:** Because it's convenient to drink caffeinated beverages
**CMQ_10:** To help me focus my attention
**CMQ_11:** Because I like the "jolt" of energy rush that I feel
**CMQ_12:** To help me relax or calm down
**CMQ_13:** To stay awake
**CMQ_14:** As a social pastime
**CMQ_15:** Because it is an ingredient in my diet pills
**CMQ_16:** Because I crave caffeine
**CMQ_17:** As a reward to myself for completing a task
**CMQ_18:** Seeing others ingest caffeine makes me crave it
**CMQ_19:** Because it is a powerful diuretic
**CMQ_20:** Because it puts me in a better mood
**CMQ_21:** To help lose or control my weight

Table S2. Items of CUDQ-10

**CUDQ_01:** Did you feel a strong desire or have unsuccessful attempts to reduce or control your caffeine consumption?
**CUDQ_02:** Did you consume caffeine even though you knew it could cause permanent or recurrent physical or psychological consequences?
**CUDQ_03:** Did you consume caffeine in order to avoid one or more caffeine withdrawal symptoms (e.g., headache, nausea, fatigue)?
**CUDQ_04:** Did you consume more caffeine, or did you consume caffeine for a longer period than you intended?
**CUDQ_05:** Because of caffeine use, did you fail to fulfill any major work, school, or home responsibilities (e.g., repeated absences from work or school due to caffeine consumption or withdrawal symptoms)?
**CUDQ_06:** Did you consume caffeine even though you knew it could cause permanent or recurrent social problems or exacerbate them (e.g., arguments with spouse because of the consequences, medical problems and costs due to caffeine use)?
**CUDQ_07:** Did you have to consume more caffeine than before to reach the same effect, or did you experience that the same amount of caffeine did not have the desired effect anymore?

CUDQ_08: Did you spend a significant amount of time consuming or obtaining caffeine?
CUDQ_09: Did you feel a strong desire or urge to consume caffeine?
CUDQ_10: Did the above-mentioned phenomena, which you experienced, cause you significant inconvenience or suffering in your everyday life?

Table S3. MOS-Sleep Scale

MOSS_01: usual time to fall asleep.
MOSS_02: average hours of sleep each night.
MOSS_03: frequency of feeling sleep was not restful.
MOSS_04: frequency of feeling sufficiently rested upon waking up.
MOSS_05: frequency of waking up with shortness of breath or a headache.
MOSS_06: frequency of feeling sleepy or drowsy during the day.
MOSS_07: frequency of having trouble falling asleep
MOSS_08: frequency of waking up during sleep and having trouble going back to sleep.
MOSS_09: frequency of having difficulty staying awake during the day.
MOSS_10: frequency of snoring during sleep.
MOSS_11: frequency of taking naps or falling asleep during the day.
MOSS_12: frequency of getting the amount of sleep you needed.

Table S4. PHQ-9 Items

PHQ_01: Little interest or pleasure in doing things
PHQ_02: Feeling down, depressed, or hopeless
PHQ_03: Trouble falling or staying asleep, or sleeping too much
PHQ_04: Feeling tired or having little energy
PHQ_05: Poor appetite or overeating
PHQ_06: Feeling bad about yourself — or that you are a failure or have let yourself or your family down
PHQ_07: Trouble concentrating on things, such as reading the newspaper or watching television
PHQ_08: Moving or speaking so slowly that other people could have noticed? Or the opposite — being so fidgety or restless that you have been moving around a lot more than usual
PHQ_09: Thoughts that you would be better off dead or of hurting yourself in some way

Table S5. All codes and outputs of the analysis

```
Data <- read_spss("D:/B_Amin/Caffine/Received files/Data_R.sav")
>
> ##-----------------Subset of data (Caffeine consumers)
>
> Subset <- Data %>%
+   filter(Caffeine_Cons == 1) %>%
+   select(CUDQ_01:CUDQ_10,
+          CMQ_01:CMQ_21,
+          PHQ_01:PHQ_09,
+          MOSS_01, MOSS_03:MOSS_12)
> Subset
# A tibble: 383 × 51
   CUDQ_01     CUDQ_02 CUDQ_03 CUDQ_04 CUDQ_05 CUDQ_06 CUDQ_07 CUDQ_08 CUDQ_09 CUDQ
_10 CMQ_01  CMQ_02  CMQ_03  CMQ_04  CMQ_05  CMQ_06  CMQ_07  CMQ_08  CMQ_09  CMQ_10 
CMQ_11  CMQ_12
   <dbl+lbl>   <dbl+l> <dbl+l> <dbl+l> <dbl+l> <dbl+l> <dbl+l> <dbl+l> <dbl+l> <dbl
+l> <dbl+l> <dbl+l> <dbl+l> <dbl+l> <dbl+l> <dbl+l> <dbl+l> <dbl+l> <dbl+l> <dbl+l>
<dbl+l> <dbl+l>
 1 0 [Never]   1 [Som… 0 [Nev… 0 [Nev… 0 [Nev… 0 [Nev… 0 [Nev… 0 [Nev… 0 [Nev… 0 [N
ev… 2 [Rar… 4 [Oft… 1 [Nev… 1 [Nev… 1 [Nev… 1 [Nev… 1 [Nev… 1 [Nev… 1 [Nev… 1 [Nev…
2 [Rar… 1 [Nev…
 2 2 [Often]   3 [Alw… 1 [Som… 3 [Alw… 0 [Nev… 0 [Nev… 0 [Nev… 1 [Som… 2 [Oft… 0 [N
ev… 1 [Nev… 2 [Rar… 1 [Nev… 5 [Alw… 1 [Nev… 2 [Rar… 2 [Rar… 1 [Nev… 3 [Som… 1 [Nev…
4 [Oft… 5 [Alw…
 3 0 [Never]   1 [Som… 0 [Nev… 0 [Nev… 0 [Nev… 0 [Nev… 0 [Nev… 0 [Nev… 1 [Som… 0 [N
ev… 2 [Rar… 2 [Rar… 2 [Rar… 3 [Som… 2 [Rar… 1 [Nev… 1 [Nev… 3 [Som… 1 [Nev… 3 [Som…
3 [Som… 2 [Rar…
 4 2 [Often]   2 [Oft… 2 [Oft… 1 [Som… 1 [Som… 1 [Som… 1 [Som… 2 [Oft… 2 [Oft… 2 [O
ft… 2 [Rar… 4 [Oft… 4 [Oft… 3 [Som… 3 [Som… 3 [Som… 2 [Rar… 4 [Oft… 4 [Oft… 3 [Som…
4 [Oft… 3 [Som…
 5 2 [Often]   2 [Oft… 2 [Oft… 1 [Som… 1 [Som… 1 [Som… 1 [Som… 2 [Oft… 2 [Oft… 2 [O
ft… 2 [Rar… 3 [Som… 3 [Som… 2 [Rar… 2 [Rar… 2 [Rar… 1 [Nev… 3 [Som… 3 [Som… 2 [Rar…
3 [Som… 2 [Rar…
 6 1 [Sometim… 1 [Som… 1 [Som… 0 [Nev… 0 [Nev… 0 [Nev… 0 [Nev… 0 [Nev… 1 [Som… 1 [S
om… 3 [Som… 3 [Som… 2 [Rar… 2 [Rar… 2 [Rar… 1 [Nev… 1 [Nev… 3 [Som… 2 [Rar… 2 [Rar…
3 [Som… 2 [Rar…
 7 0 [Never]   3 [Alw… 2 [Oft… 0 [Nev… 0 [Nev… 1 [Som… 0 [Nev… 0 [Nev… 1 [Som… 0 [N
ev… 5 [Alw… 3 [Som… 4 [Oft… 1 [Nev… 2 [Rar… 1 [Nev… 1 [Nev… 5 [Alw… 3 [Som… 2 [Rar…
5 [Alw… 5 [Alw…
 8 1 [Sometim… 2 [Oft… 1 [Som… 2 [Oft… 0 [Nev… 1 [Som… 2 [Oft… 2 [Oft… 3 [Alw… 1 [S
om… 4 [Oft… 5 [Alw… 4 [Oft… 5 [Alw… 2 [Rar… 2 [Rar… 2 [Rar… 5 [Alw… 4 [Oft… 4 [Oft…
5 [Alw… 5 [Alw…
 9 0 [Never]   2 [Oft… 1 [Som… 0 [Nev… 0 [Nev… 1 [Som… 0 [Nev… 1 [Som… 0 [Nev… 1 [S
om… 2 [Rar… 2 [Rar… 3 [Som… 2 [Rar… 2 [Rar… 2 [Rar… 3 [Som… 3 [Som… 3 [Som… 3 [Som…
3 [Som… 3 [Som…
10 0 [Never]   2 [Oft… 1 [Som… 1 [Som… 0 [Nev… 0 [Nev… 0 [Nev… 1 [Som… 0 [Nev… 0 [N
ev… 2 [Rar… 3 [Som… 3 [Som… 3 [Som… 2 [Rar… 2 [Rar… 1 [Nev… 3 [Som… 3 [Som… 3 [Som…
2 [Rar… 2 [Rar…
# ℹ 373 more rows
# ℹ 29 more variables: CMQ_13 <dbl+lbl>, CMQ_14 <dbl+lbl>, CMQ_15 <dbl+lbl>, CMQ_16
<dbl+lbl>, CMQ_17 <dbl+lbl>, CMQ_18 <dbl+lbl>, CMQ_19 <dbl+lbl>, CMQ_20 <dbl+lbl>,
```

```
#   CMQ_21 <dbl+lbl>, PHQ_01 <dbl+lbl>, PHQ_02 <dbl+lbl>, PHQ_03 <dbl+lbl>, PHQ_04
<dbl+lbl>, PHQ_05 <dbl+lbl>, PHQ_06 <dbl+lbl>, PHQ_07 <dbl+lbl>, PHQ_08 <dbl+lbl>,
#   PHQ_09 <dbl+lbl>, MOSS_01 <dbl>, MOSS_03 <dbl>, MOSS_04 <dbl>, MOSS_05 <dbl>, M
OSS_06 <dbl>, MOSS_07 <dbl>, MOSS_08 <dbl>, MOSS_09 <dbl>, MOSS_10 <dbl>, MOSS_11 <
dbl>,
#   MOSS_12 <dbl>
# ℹ Use `print(n = ...)` to see more rows
>
> ##-----------------Creating a coefficient matrix applicable for analysis (Non-par
a[For likert])
> Cov_mat <- cor(Subset, use = "pairwise.complete.obs", method = "kendall")
> rownames(Cov_mat) <- colnames(Cov_mat)
>
>
>
> ##-----------------Domains of the Study as groups
> groups <- list(
+   Caffeine_Use_Disorder = paste0("CUDQ_", sprintf("%02d", 1:10)),
+   Caffeine_Motive = paste0("CMQ_", sprintf("%02d", 1:21)),
+   Depression = paste0("PHQ_", sprintf("%02d", 1:9)),
+   MOS_Sleep = c("MOS_01", paste0("MOS_", sprintf("%02d", 3:12)))
+ )
>
> community_colors <- c(
+   "Caffeine_Use_Disorder" = "#1f77b4",
+   "Depression" = "#ff7f0e",
+   "Caffeine_Motive" = "#2ca02c",
+   "MOS_Sleep" = "#8c564b"
+ )
>
>
> ##-----------------Designing of the network
> node_colors <- unlist(lapply(names(groups), function(g) rep(community_colors[g],
length(groups[[g]]))))
> edge_colors <- matrix(NA, nrow = ncol(Subset), ncol = ncol(Subset))
> for(i in 1:ncol(Subset)) {
+   for(j in 1:ncol(Subset)) {
+     if(i != j & Cov_mat[i,j] != 0) {
+       edge_colors[i,j] <- node_colors[i]
+     } else {
+       edge_colors[i,j] <- NA
+     }
+   }
+ }
>
>
> png("D:/B_Amin/Caffine/Caffeine_Network.png",
+     width = 6000, height = 5000, res = 300)
> network <- qgraph(Cov_mat,
+                   graph = "glasso",
+                   layout = "spring",
+                   groups = groups,
+                   sampleSize = nrow(Subset),
+                   theme = "colorblind",
+                   legend = FALSE,
+                   vsize = 7,
```

```
+                   esize = 25,
+                   edge.color = edge_colors,
+                   color = node_colors,
+                   label.cex = 1.5,
+                   label.scale = FALSE,
+                   tuning = 0.2   # smaller = presence of weaker edges
+ )
> dev.off()
png
  2
>
> ##-----------------The bridge centrality indices
>
>
> community_vector <- rep(names(groups), sapply(groups, length))
> bridge_results <- bridge(network, communities = community_vector)
> summary(bridge_results)
        Bridge.Strength Bridge.Betweenness Bridge.Closeness Bridge.Expected.Influen
ce..1.step. Bridge.Expected.Influence..2.step.           communities
CUDQ_01      0.05030235                  0       0.02007738
0.05030235                      0.126027369 Caffeine_Use_Disorder
CUDQ_02      0.27493828                 20       0.02876308
0.27493828                      0.536881108 Caffeine_Use_Disorder
CUDQ_03      0.28578385                142       0.03457755
0.28578385                      0.602976402 Caffeine_Use_Disorder
CUDQ_04      0.15541655                105       0.03356753
0.15541655                      0.434041663 Caffeine_Use_Disorder
CUDQ_05      0.22750379                 47       0.03017366
0.22750379                      0.493620335 Caffeine_Use_Disorder
CUDQ_06      0.12319852                  1       0.02689723
0.12319852                      0.319372946 Caffeine_Use_Disorder
CUDQ_07      0.29753791                117       0.03269148
0.29753791                      0.674840708 Caffeine_Use_Disorder
CUDQ_08      0.16110137                  0       0.02758758
0.16110137                      0.420578348 Caffeine_Use_Disorder
CUDQ_09      0.27634418                 62       0.03078775
0.27634418                      0.641885117 Caffeine_Use_Disorder
CUDQ_1       0.19576238                  5       0.02742386
0.19576238                      0.424955880 Caffeine_Use_Disorder
CMQ_01       0.10312149                 46       0.02832528
0.10312149                      0.221348681       Caffeine_Motive
CMQ_02       0.27252891                248       0.03217812
0.27252891                      0.453336249       Caffeine_Motive
CMQ_03       0.00117645                 57       0.02848100
0.00117645                      0.090793465       Caffeine_Motive
CMQ_04       0.20780906                 16       0.02928624
0.20780906                      0.366662991       Caffeine_Motive
CMQ_05       0.00000000                 32       0.02671858
0.00000000                      0.073114760       Caffeine_Motive
CMQ_06       0.05322101                 40       0.02679134
0.05322101                      0.148214842       Caffeine_Motive
CMQ_07       0.06023525                  6       0.02550243
0.06023525                      0.173779197       Caffeine_Motive
CMQ_08       0.01017363                  0       0.02467773
0.01017363                      0.076177486       Caffeine_Motive
```

CMQ_09	0.08920462	45	0.02672374	0.08920462	0.213518389	Caffeine_Motive
CMQ_10	0.01784401	17	0.02521334	0.01784401	0.061471168	Caffeine_Motive
CMQ_11	0.04669589	22	0.02518379	0.04669589	0.157225218	Caffeine_Motive
CMQ_12	0.08515280	9	0.02368326	0.08515280	0.173312635	Caffeine_Motive
CMQ_13	0.04510829	19	0.02513369	0.04510829	0.125276408	Caffeine_Motive
CMQ_14	0.05384615	6	0.02450509	0.05384615	0.172850746	Caffeine_Motive
CMQ_15	0.24036212	121	0.03142264	0.24036212	0.533285452	Caffeine_Motive
CMQ_16	0.31757389	63	0.02970874	0.31757389	0.670536822	Caffeine_Motive
CMQ_17	0.25648989	86	0.03142437	0.25648989	0.578974432	Caffeine_Motive
CMQ_18	0.19464439	39	0.02942542	0.19464439	0.438130577	Caffeine_Motive
CMQ_19	0.19503799	5	0.02900752	0.19503799	0.412880624	Caffeine_Motive
CMQ_20	0.02797870	7	0.02582912	0.02797870	0.131171500	Caffeine_Motive
CMQ_21	0.19455810	113	0.03147811	0.19455810	0.427561889	Caffeine_Motive
PHQ_01	0.03403219	12	0.02614818	0.03403219	0.145258077	Depression
PHQ_02	0.10154086	33	0.02597449	0.10154086	0.273559347	Depression
PHQ_03	0.22804873	58	0.02691356	0.22804873	0.391563773	Depression
PHQ_04	0.07625612	33	0.02618981	0.07625612	0.250016094	Depression
PHQ_05	0.09520768	4	0.02375690	0.09520768	0.231633796	Depression
PHQ_06	0.18864174	143	0.03055560	0.18864174	0.407077970	Depression
PHQ_07	0.16131895	92	0.02912396	0.16131895	0.364161856	Depression
PHQ_08	0.06091358	31	0.02567822	0.06091358	0.232845077	Depression
PHQ_09	0.19436727	12	0.02491639	0.19436727	0.364175207	Depression
MOSS_01	0.04354594	0	0.02083912	0.04354594	0.095907482	MOS_Sleep
MOSS_03	0.22918427	52	0.02775507	0.22918427	0.419928471	MOS_Sleep
MOSS_04	0.00000000	0	0.01541315	0.00000000	0.006232546	MOS_Sleep
MOSS_05	0.15589153	66	0.02912608	0.15589153	0.380962359	MOS_Sleep
MOSS_06	0.11629640	7	0.02570268	0.11629640	0.267963405	MOS_Sleep
MOSS_07	0.10521797	51	0.02762695	0.10521797	0.289574023	MOS_Sleep

```
MOSS_08      0.06417670                  29      0.02672383
0.06417670                       0.208010116           MOS_Sleep
MOSS_09      0.20179514                  47      0.02828594
0.20179514                       0.421505529           MOS_Sleep
MOSS_10      0.27004146                 118      0.03044777
0.27004146                       0.477623645           MOS_Sleep
MOSS_11      0.00000000                   0      0.02624880
0.00000000                       0.070784914           MOS_Sleep
MOSS_12      0.06117004                  40      0.01857541
0.06117004                       0.107084716           MOS_Sleep
>
> png("D:/B_Amin/Caffine/Bridge_strength.png",
+     width = 4000, height = 5000, res = 300)
> plot(bridge_results)
Warning message:
Vectorized input to `element_text()` is not officially supported.
i Results may be unexpected or may change in future versions of ggplot2. > dev.off(
)
png
  2
>
>
>
> ##-----------------Top 10 bridges for reporting (Convenience and self style)
> top_bridges <- sort(bridge_results$`Bridge Strength`, decreasing = TRUE)
> print(head(top_bridges, 10))
   CMQ_16   CUDQ_07   CUDQ_03   CUDQ_09   CUDQ_02    CMQ_02   MOSS_10    CMQ_17
CMQ_15   MOSS_03
0.3175739 0.2975379 0.2857839 0.2763442 0.2749383 0.2725289 0.2700415 0.2564899 0.2
403621 0.2291843
>
>
>
>
> ############################## SEM analysis
>
>
> #--------------------Computing SUM------------------------#
>
> library(lavaan)
> library(dplyr)
>
>
> #--------------------Conceptual SEM------------------------#
>
> model_items <- '
+   # Latent variables
+   CUDQ =~ CUDQ_01 + CUDQ_02 + CUDQ_03 + CUDQ_04 + CUDQ_05 + CUDQ_06 + CUDQ_07 + C
UDQ_08 + CUDQ_09 + CUDQ_10
+   CMQ  =~ CMQ_01 + CMQ_02 + CMQ_03 + CMQ_04 + CMQ_05 + CMQ_06 + CMQ_07 + CMQ_08 +
CMQ_09 + CMQ_10
+          + CMQ_11 + CMQ_12 + CMQ_13 + CMQ_14 + CMQ_15 + CMQ_16 + CMQ_17 + CMQ_18
+ CMQ_19 + CMQ_20 + CMQ_21
+   PHQ  =~ PHQ_01 + PHQ_02 + PHQ_03 + PHQ_04 + PHQ_05 + PHQ_06 + PHQ_07 + PHQ_08 +
PHQ_09
```

```
+   MOS  =~ MOSS_01 + MOSS_03 + MOSS_04 + MOSS_05 + MOSS_06 + MOSS_07 + MOSS_08 + M
OSS_09 + MOSS_10 + MOSS_11 + MOSS_12
+
+   # Structural paths
+   CMQ ~ a*CUDQ       # CUDQ -> CMQ
+   PHQ ~ b*CUDQ       # CUDQ -> PHQ
+   MOS ~ c*CUDQ + d*CMQ + e*PHQ   # Direct effects on MOS
+
+   # Indirect effects
+   indirect_CUDQ_CM_MOS := a * d   # CUDQ -> CMQ -> MOS
+   indirect_CUDQ_PH_MOS := b * e   # CUDQ -> PHQ -> MOS
+   total_CUDQ_MOS := c + (a*d) + (b*e)  # Total effect
+ '
>
>
> fit_items <- sem(model_items, data = Subset, estimator = "DWLS")
>
>
> summary(fit_items, standardized = TRUE, fit.measures = TRUE, rsquare = TRUE, ci =
TRUE)
lavaan 0.6-18 ended normally after 94 iterations

  Estimator                                         DWLS
  Optimization method                             NLMINB
  Number of model parameters                         158

  Number of observations                             383
  Number of missing patterns                           1

Model Test User Model:

  Test statistic                                3625.176
  Degrees of freedom                                1219
  P-value (Chi-square)                             0.000

Model Test Baseline Model:

  Test statistic                                8862.600
  Degrees of freedom                                1275
  P-value                                          0.000

User Model versus Baseline Model:

  Comparative Fit Index (CFI)                      0.683
  Tucker-Lewis Index (TLI)                         0.668

  Robust Comparative Fit Index (CFI)               0.683
  Robust Tucker-Lewis Index (TLI)                  0.668

Loglikelihood and Information Criteria:

  Loglikelihood user model (H0)               -28174.773
  Loglikelihood unrestricted model (H1)               NA

  Akaike (AIC)                                 56665.545
  Bayesian (BIC)                               57289.335
```

```
  Sample-size adjusted Bayesian (SABIC)      56788.025

Root Mean Square Error of Approximation:

  RMSEA                                          0.072
  90 Percent confidence interval - lower         0.069
  90 Percent confidence interval - upper         0.074
  P-value H_0: RMSEA <= 0.050                    0.000
  P-value H_0: RMSEA >= 0.080                    0.000

  Robust RMSEA                                   0.072
  90 Percent confidence interval - lower         0.069
  90 Percent confidence interval - upper         0.074
  P-value H_0: Robust RMSEA <= 0.050             0.000
  P-value H_0: Robust RMSEA >= 0.080             0.000

Standardized Root Mean Square Residual:

  SRMR                                           0.083

Parameter Estimates:

  Standard errors                             Standard
  Information                                 Observed
  Observed information based on                Hessian

Latent Variables:
                   Estimate  Std.Err  z-value  P(>|z|) ci.lower ci.upper   Std.lv
Std.all
  CUDQ =~
    CUDQ_01           1.000                               1.000    1.000    0.240
0.273
    CUDQ_02           1.777    0.421    4.223    0.000    0.952    2.602    0.427
0.410
    CUDQ_03           1.782    0.405    4.402    0.000    0.989    2.576    0.428
0.451
    CUDQ_04           1.946    0.411    4.734    0.000    1.140    2.752    0.468
0.603
    CUDQ_05           1.089    0.241    4.514    0.000    0.616    1.562    0.262
0.477
    CUDQ_06           1.618    0.364    4.439    0.000    0.904    2.332    0.389
0.481
    CUDQ_07           2.003    0.420    4.767    0.000    1.180    2.827    0.481
0.658
    CUDQ_08           1.830    0.396    4.622    0.000    1.054    2.606    0.440
0.583
    CUDQ_09           2.647    0.552    4.795    0.000    1.565    3.728    0.636
0.705
    CUDQ_10           1.439    0.326    4.411    0.000    0.800    2.078    0.346
0.449
  CMQ =~
    CMQ_01            1.000                               1.000    1.000    0.547
0.451
    CMQ_02            1.226    0.163    7.544    0.000    0.908    1.545    0.671
0.536
```

```
    CMQ_03            1.261    0.162    7.765    0.000    0.943    1.580    0.690
0.578
    CMQ_04            1.314    0.177    7.430    0.000    0.968    1.661    0.719
0.540
    CMQ_05            1.709    0.202    8.476    0.000    1.314    2.104    0.935
0.725
    CMQ_06            1.628    0.193    8.419    0.000    1.249    2.007    0.891
0.719
    CMQ_07            1.545    0.186    8.300    0.000    1.180    1.910    0.845
0.694
    CMQ_08            1.363    0.175    7.779    0.000    1.020    1.707    0.746
0.580
    CMQ_09            1.512    0.185    8.170    0.000    1.150    1.875    0.827
0.645
    CMQ_10            1.496    0.181    8.256    0.000    1.141    1.851    0.819
0.676
    CMQ_11            1.648    0.199    8.268    0.000    1.257    2.039    0.902
0.670
    CMQ_12            1.505    0.190    7.938    0.000    1.134    1.877    0.823
0.607
    CMQ_13            1.339    0.174    7.677    0.000    0.997    1.681    0.732
0.556
    CMQ_14            1.341    0.180    7.445    0.000    0.988    1.693    0.733
0.536
    CMQ_15            1.127    0.156    7.205    0.000    0.821    1.434    0.617
0.523
    CMQ_16            1.362    0.175    7.763    0.000    1.018    1.706    0.745
0.606
    CMQ_17            1.347    0.172    7.812    0.000    1.009    1.685    0.737
0.616
    CMQ_18            1.449    0.187    7.746    0.000    1.083    1.816    0.793
0.600
    CMQ_19            0.782    0.133    5.866    0.000    0.521    1.044    0.428
0.377
    CMQ_20            1.404    0.183    7.675    0.000    1.046    1.763    0.768
0.590
    CMQ_21            0.663    0.123    5.373    0.000    0.421    0.905    0.363
0.335
  PHQ =~
    PHQ_01            1.000                               1.000    1.000    0.655
0.618
    PHQ_02            1.101    0.099   11.133    0.000    0.907    1.295    0.721
0.696
    PHQ_03            0.999    0.097   10.268    0.000    0.809    1.190    0.654
0.639
    PHQ_04            1.007    0.097   10.399    0.000    0.817    1.197    0.659
0.659
    PHQ_05            0.852    0.097    8.769    0.000    0.661    1.042    0.558
0.537
    PHQ_06            1.188    0.108   10.982    0.000    0.976    1.400    0.778
0.697
    PHQ_07            1.131    0.104   10.926    0.000    0.928    1.334    0.741
0.696
    PHQ_08            1.011    0.097   10.392    0.000    0.821    1.202    0.662
0.665
```

```
    PHQ_09            0.876    0.097    9.057    0.000    0.686    1.065    0.573
0.556
  MOS =~
    MOSS_01           1.000                               1.000    1.000    0.473
0.308
    MOSS_03           1.844    0.354    5.205    0.000    1.149    2.538    0.871
0.555
    MOSS_04          -0.089    0.184   -0.483    0.629   -0.450    0.272   -0.042
-0.028
    MOSS_05           2.227    0.427    5.220    0.000    1.391    3.063    1.052
0.627
    MOSS_06           1.523    0.311    4.904    0.000    0.914    2.132    0.720
0.494
    MOSS_07           2.531    0.466    5.431    0.000    1.618    3.445    1.196
0.723
    MOSS_08           2.417    0.443    5.462    0.000    1.550    3.285    1.142
0.703
    MOSS_09           2.068    0.398    5.195    0.000    1.288    2.848    0.977
0.599
    MOSS_10           1.918    0.387    4.956    0.000    1.160    2.677    0.907
0.505
    MOSS_11           1.555    0.322    4.832    0.000    0.925    2.186    0.735
0.448
    MOSS_12          -0.106    0.173   -0.611    0.541   -0.446    0.234   -0.050
-0.035

Regressions:
                   Estimate  Std.Err  z-value  P(>|z|) ci.lower ci.upper   Std.lv
Std.all
  CMQ ~
    CUDQ       (a)    1.415    0.336    4.207    0.000    0.756    2.075    0.622
0.622
  PHQ ~
    CUDQ       (b)    1.021    0.261    3.906    0.000    0.509    1.533    0.375
0.375
  MOS ~
    CUDQ       (c)    0.608    0.225    2.709    0.007    0.168    1.048    0.309
0.309
    CMQ        (d)   -0.024    0.065   -0.367    0.713   -0.151    0.103   -0.028
-0.028
    PHQ        (e)    0.221    0.061    3.647    0.000    0.102    0.340    0.306
0.306

Intercepts:
                   Estimate  Std.Err  z-value  P(>|z|) ci.lower ci.upper   Std.lv
Std.all
   .CUDQ_01           0.614    0.045   13.640    0.000    0.525    0.702    0.614
0.697
   .CUDQ_02           1.238    0.053   23.257    0.000    1.133    1.342    1.238
1.188
   .CUDQ_03           0.854    0.049   17.584    0.000    0.759    0.949    0.854
0.899
   .CUDQ_04           0.436    0.040   11.007    0.000    0.358    0.514    0.436
0.562
   .CUDQ_05           0.209    0.028    7.451    0.000    0.154    0.264    0.209
0.381
```

.CUDQ_06	0.590	0.041	14.275	0.000	0.509	0.671	0.590	0.729
.CUDQ_07	0.410	0.037	10.975	0.000	0.337	0.483	0.410	0.561
.CUDQ_08	0.517	0.039	13.421	0.000	0.441	0.592	0.517	0.686
.CUDQ_09	0.728	0.046	15.801	0.000	0.638	0.819	0.728	0.807
.CUDQ_10	0.465	0.039	11.807	0.000	0.388	0.542	0.465	0.603
.CMQ_01	2.475	0.062	39.935	0.000	2.354	2.597	2.475	2.041
.CMQ_02	2.668	0.064	41.692	0.000	2.543	2.794	2.668	2.130
.CMQ_03	2.580	0.061	42.309	0.000	2.460	2.699	2.580	2.162
.CMQ_04	2.713	0.068	39.891	0.000	2.580	2.846	2.713	2.038
.CMQ_05	2.287	0.066	34.724	0.000	2.158	2.416	2.287	1.774
.CMQ_06	2.285	0.063	36.085	0.000	2.161	2.409	2.285	1.844
.CMQ_07	2.167	0.062	34.847	0.000	2.045	2.289	2.167	1.781
.CMQ_08	2.786	0.066	42.361	0.000	2.657	2.915	2.786	2.165
.CMQ_09	2.501	0.066	38.175	0.000	2.373	2.630	2.501	1.951
.CMQ_10	2.512	0.062	40.590	0.000	2.390	2.633	2.512	2.074
.CMQ_11	2.760	0.069	40.126	0.000	2.625	2.895	2.760	2.050
.CMQ_12	2.611	0.069	37.661	0.000	2.475	2.747	2.611	1.924
.CMQ_13	2.736	0.067	40.659	0.000	2.604	2.868	2.736	2.078
.CMQ_14	2.595	0.070	37.141	0.000	2.458	2.732	2.595	1.898
.CMQ_15	1.830	0.060	30.393	0.000	1.712	1.948	1.830	1.553
.CMQ_16	1.950	0.063	31.016	0.000	1.827	2.074	1.950	1.585
.CMQ_17	1.953	0.061	31.978	0.000	1.833	2.073	1.953	1.634
.CMQ_18	2.164	0.068	32.060	0.000	2.032	2.297	2.164	1.638
.CMQ_19	1.755	0.058	30.268	0.000	1.641	1.868	1.755	1.547
.CMQ_20	2.243	0.067	33.682	0.000	2.112	2.373	2.243	1.721
.CMQ_21	1.718	0.055	31.034	0.000	1.610	1.827	1.718	1.586
.PHQ_01	2.214	0.054	40.873	0.000	2.108	2.320	2.214	2.089
.PHQ_02	2.172	0.053	41.058	0.000	2.069	2.276	2.172	2.098

```
   .PHQ_03           2.204    0.052   42.089    0.000    2.101    2.306    2.204
2.151
   .PHQ_04           2.175    0.051   42.549    0.000    2.075    2.275    2.175
2.174
   .PHQ_05           2.089    0.053   39.366    0.000    1.985    2.193    2.089
2.012
   .PHQ_06           2.175    0.057   38.129    0.000    2.063    2.287    2.175
1.948
   .PHQ_07           2.214    0.054   40.685    0.000    2.107    2.321    2.214
2.079
   .PHQ_08           1.896    0.051   37.252    0.000    1.796    1.995    1.896
1.903
   .PHQ_09           1.794    0.053   34.066    0.000    1.691    1.897    1.794
1.741
   .MOSS_01          1.569    0.079   19.988    0.000    1.415    1.723    1.569
1.021
   .MOSS_03          2.548    0.080   31.743    0.000    2.391    2.706    2.548
1.622
   .MOSS_04          2.178    0.078   27.925    0.000    2.025    2.330    2.178
1.427
   .MOSS_05          1.770    0.086   20.645    0.000    1.602    1.938    1.770
1.055
   .MOSS_06          2.726    0.075   36.580    0.000    2.580    2.872    2.726
1.869
   .MOSS_07          2.366    0.085   27.963    0.000    2.200    2.531    2.366
1.429
   .MOSS_08          1.883    0.083   22.680    0.000    1.720    2.045    1.883
1.159
   .MOSS_09          2.420    0.083   29.026    0.000    2.257    2.584    2.420
1.483
   .MOSS_10          1.431    0.092   15.610    0.000    1.251    1.610    1.431
0.798
   .MOSS_11          2.360    0.084   28.159    0.000    2.196    2.525    2.360
1.439
   .MOSS_12          2.016    0.073   27.468    0.000    1.872    2.159    2.016
1.404

Variances:
                   Estimate  Std.Err  z-value  P(>|z|) ci.lower ci.upper   Std.lv
Std.all
   .CUDQ_01          0.717    0.053   13.607    0.000    0.614    0.821    0.717
0.926
   .CUDQ_02          0.902    0.068   13.191    0.000    0.768    1.036    0.902
0.832
   .CUDQ_03          0.720    0.055   13.093    0.000    0.612    0.827    0.720
0.797
   .CUDQ_04          0.382    0.031   12.152    0.000    0.321    0.444    0.382
0.636
   .CUDQ_05          0.233    0.018   12.933    0.000    0.197    0.268    0.233
0.772
   .CUDQ_06          0.503    0.039   12.949    0.000    0.427    0.579    0.503
0.769
   .CUDQ_07          0.303    0.026   11.591    0.000    0.252    0.354    0.303
0.567
   .CUDQ_08          0.375    0.030   12.382    0.000    0.316    0.434    0.375
0.660
```

.CUDQ_09	0.410	0.037	10.926	0.000	0.336	0.483	0.410	0.503
.CUDQ_10	0.474	0.036	13.094	0.000	0.403	0.545	0.474	0.799
.CMQ_01	1.172	0.087	13.505	0.000	1.002	1.342	1.172	0.797
.CMQ_02	1.119	0.084	13.308	0.000	0.954	1.283	1.119	0.713
.CMQ_03	0.948	0.072	13.206	0.000	0.807	1.088	0.948	0.666
.CMQ_04	1.254	0.094	13.362	0.000	1.070	1.438	1.254	0.708
.CMQ_05	0.787	0.063	12.461	0.000	0.663	0.911	0.787	0.474
.CMQ_06	0.742	0.059	12.485	0.000	0.625	0.858	0.742	0.483
.CMQ_07	0.767	0.060	12.720	0.000	0.649	0.885	0.767	0.518
.CMQ_08	1.100	0.083	13.211	0.000	0.937	1.263	1.100	0.664
.CMQ_09	0.960	0.074	12.995	0.000	0.815	1.104	0.960	0.584
.CMQ_10	0.797	0.062	12.849	0.000	0.675	0.918	0.797	0.543
.CMQ_11	0.999	0.078	12.879	0.000	0.847	1.151	0.999	0.551
.CMQ_12	1.163	0.089	13.121	0.000	0.989	1.336	1.163	0.632
.CMQ_13	1.198	0.090	13.265	0.000	1.021	1.375	1.198	0.691
.CMQ_14	1.332	0.100	13.342	0.000	1.137	1.528	1.332	0.712
.CMQ_15	1.009	0.075	13.363	0.000	0.861	1.157	1.009	0.726
.CMQ_16	0.959	0.073	13.070	0.000	0.815	1.103	0.959	0.633
.CMQ_17	0.886	0.068	13.018	0.000	0.752	1.019	0.886	0.620
.CMQ_18	1.117	0.085	13.122	0.000	0.950	1.284	1.117	0.640
.CMQ_19	1.104	0.081	13.626	0.000	0.945	1.263	1.104	0.858
.CMQ_20	1.108	0.084	13.200	0.000	0.943	1.272	1.108	0.652
.CMQ_21	1.042	0.076	13.675	0.000	0.893	1.191	1.042	0.888
.PHQ_01	0.695	0.056	12.514	0.000	0.586	0.804	0.695	0.619
.PHQ_02	0.552	0.047	11.792	0.000	0.460	0.644	0.552	0.515
.PHQ_03	0.622	0.051	12.278	0.000	0.522	0.721	0.622	0.592
.PHQ_04	0.566	0.047	12.154	0.000	0.475	0.657	0.566	0.566
.PHQ_05	0.767	0.059	12.956	0.000	0.651	0.883	0.767	0.712

```
   .PHQ_06            0.642    0.055   11.742    0.000    0.534    0.749    0.642
0.515
   .PHQ_07            0.586    0.050   11.723    0.000    0.488    0.684    0.586
0.516
   .PHQ_08            0.553    0.046   12.088    0.000    0.463    0.643    0.553
0.558
   .PHQ_09            0.733    0.057   12.856    0.000    0.621    0.845    0.733
0.690
   .MOSS_01           2.137    0.158   13.532    0.000    1.828    2.447    2.137
0.905
   .MOSS_03           1.709    0.136   12.545    0.000    1.442    1.976    1.709
0.692
   .MOSS_04           2.327    0.168   13.836    0.000    1.997    2.657    2.327
0.999
   .MOSS_05           1.709    0.144   11.845    0.000    1.426    1.991    1.709
0.607
   .MOSS_06           1.609    0.125   12.838    0.000    1.363    1.854    1.609
0.756
   .MOSS_07           1.310    0.123   10.669    0.000    1.069    1.551    1.310
0.478
   .MOSS_08           1.334    0.121   11.007    0.000    1.096    1.571    1.334
0.506
   .MOSS_09           1.708    0.141   12.106    0.000    1.432    1.985    1.708
0.642
   .MOSS_10           2.396    0.188   12.714    0.000    2.027    2.766    2.396
0.745
   .MOSS_11           2.151    0.164   13.077    0.000    1.828    2.473    2.151
0.799
   .MOSS_12           2.060    0.149   13.834    0.000    1.768    2.352    2.060
0.999
    CUDQ              0.058    0.023    2.480    0.013    0.012    0.103    1.000
1.000
   .CMQ               0.184    0.042    4.351    0.000    0.101    0.266    0.614
0.614
   .PHQ               0.369    0.060    6.181    0.000    0.252    0.485    0.860
0.860
   .MOS               0.168    0.060    2.788    0.005    0.050    0.286    0.753
0.753

R-Square:
                   Estimate
    CUDQ_01           0.074
    CUDQ_02           0.168
    CUDQ_03           0.203
    CUDQ_04           0.364
    CUDQ_05           0.228
    CUDQ_06           0.231
    CUDQ_07           0.433
    CUDQ_08           0.340
    CUDQ_09           0.497
    CUDQ_10           0.201
    CMQ_01            0.203
    CMQ_02            0.287
    CMQ_03            0.334
    CMQ_04            0.292
    CMQ_05            0.526
```

```
    CMQ_06              0.517
    CMQ_07              0.482
    CMQ_08              0.336
    CMQ_09              0.416
    CMQ_10              0.457
    CMQ_11              0.449
    CMQ_12              0.368
    CMQ_13              0.309
    CMQ_14              0.288
    CMQ_15              0.274
    CMQ_16              0.367
    CMQ_17              0.380
    CMQ_18              0.360
    CMQ_19              0.142
    CMQ_20              0.348
    CMQ_21              0.112
    PHQ_01              0.381
    PHQ_02              0.485
    PHQ_03              0.408
    PHQ_04              0.434
    PHQ_05              0.288
    PHQ_06              0.485
    PHQ_07              0.484
    PHQ_08              0.442
    PHQ_09              0.310
    MOSS_01             0.095
    MOSS_03             0.308
    MOSS_04             0.001
    MOSS_05             0.393
    MOSS_06             0.244
    MOSS_07             0.522
    MOSS_08             0.494
    MOSS_09             0.358
    MOSS_10             0.255
    MOSS_11             0.201
    MOSS_12             0.001
    CMQ                 0.386
    PHQ                 0.140
    MOS                 0.247

Defined Parameters:
                   Estimate  Std.Err  z-value  P(>|z|) ci.lower ci.upper   Std.lv
Std.all
    in_CUDQ_CM_MOS   -0.034    0.092   -0.366    0.715   -0.215    0.147   -0.017
-0.017
    in_CUDQ_PH_MOS    0.226    0.081    2.785    0.005    0.067    0.385    0.115
0.115
    total_CUDQ_MOS    0.800    0.239    3.347    0.001    0.332    1.269    0.407
0.407

>
> # Remaining extraction values
> parameterEstimates(fit_items, standardized = TRUE) %>%
+   filter(op %in% c("~", ":="))
                   lhs op           rhs                label    est    se      z pv
alue ci.lower ci.upper std.lv std.all
```

```
1                   CMQ  ~           CUDQ                   a  1.415 0.336  4.207  0
.000    0.756    2.075  0.622   0.622
2                   PHQ  ~           CUDQ                   b  1.021 0.261  3.906  0
.000    0.509    1.533  0.375   0.375
3                   MOS  ~           CUDQ                   c  0.608 0.225  2.709  0
.007    0.168    1.048  0.309   0.309
4                   MOS  ~            CMQ                   d -0.024 0.065 -0.367  0
.713   -0.151    0.103 -0.028  -0.028
5                   MOS  ~            PHQ                   e  0.221 0.061  3.647  0
.000    0.102    0.340  0.306   0.306
6 indirect_CUDQ_CM_MOS :=             a*d indirect_CUDQ_CM_MOS -0.034 0.092 -0.366  0
.715   -0.215    0.147 -0.017  -0.017
7 indirect_CUDQ_PH_MOS :=             b*e indirect_CUDQ_PH_MOS  0.226 0.081  2.785  0
.005    0.067    0.385  0.115   0.115
8       total_CUDQ_MOS := c+(a*d)+(b*e)       total_CUDQ_MOS  0.800 0.239  3.347  0
.001    0.332    1.269  0.407   0.407
>
>
> #---------------------Modified SEM-------------------------#
>
> model_redesign <- '
+   # Latent variables
+   CUDQ =~ CUDQ_01 + CUDQ_02 + CUDQ_03 + CUDQ_04 + CUDQ_05 + CUDQ_06 + CUDQ_07 + C
UDQ_08 + CUDQ_09 + CUDQ_10
+   PHQ  =~ PHQ_01 + PHQ_02 + PHQ_03 + PHQ_04 + PHQ_05 + PHQ_06 + PHQ_07 + PHQ_08 +
PHQ_09
+   MOS  =~ MOSS_01 + MOSS_03 + MOSS_04 + MOSS_05 + MOSS_06 + MOSS_07 + MOSS_08 + M
OSS_09 + MOSS_10 + MOSS_11 + MOSS_12
+
+   # Structural paths
+   PHQ ~ b*CUDQ       # Only keep significant mediator
+   MOS ~ c*CUDQ + e*PHQ   # Only keep significant predictors
+
+   # Indirect effect
+   indirect_CUDQ_PH_MOS := b*e
+   total_CUDQ_MOS := c + (b*e)
+ '
> fit_redesign <- sem(model_redesign, data = Subset, estimator = "DWLS")
> summary(fit_redesign, standardized = TRUE, fit.measures = TRUE, rsquare = TRUE, c
i = TRUE)
lavaan 0.6-18 ended normally after 57 iterations

  Estimator                                         DWLS
  Optimization method                             NLMINB
  Number of model parameters                          93

  Number of observations                             383
  Number of missing patterns                           1

Model Test User Model:

  Test statistic                                1157.819
  Degrees of freedom                                 402
  P-value (Chi-square)                             0.000

Model Test Baseline Model:
```

```
  Test statistic                              3733.484
  Degrees of freedom                               435
  P-value                                        0.000

User Model versus Baseline Model:

  Comparative Fit Index (CFI)                    0.771
  Tucker-Lewis Index (TLI)                       0.752

  Robust Comparative Fit Index (CFI)             0.771
  Robust Tucker-Lewis Index (TLI)                0.752

Loglikelihood and Information Criteria:

  Loglikelihood user model (H0)             -16282.148
  Loglikelihood unrestricted model (H1)             NA

  Akaike (AIC)                               32750.297
  Bayesian (BIC)                             33117.464
  Sample-size adjusted Bayesian (SABIC)      32822.389

Root Mean Square Error of Approximation:

  RMSEA                                          0.070
  90 Percent confidence interval - lower         0.065
  90 Percent confidence interval - upper         0.075
  P-value H_0: RMSEA <= 0.050                    0.000
  P-value H_0: RMSEA >= 0.080                    0.000

  Robust RMSEA                                   0.070
  90 Percent confidence interval - lower         0.065
  90 Percent confidence interval - upper         0.075
  P-value H_0: Robust RMSEA <= 0.050             0.000
  P-value H_0: Robust RMSEA >= 0.080             0.000

Standardized Root Mean Square Residual:

  SRMR                                           0.072

Parameter Estimates:

  Standard errors                             Standard
  Information                                 Observed
  Observed information based on                Hessian

Latent Variables:
                   Estimate  Std.Err  z-value  P(>|z|) ci.lower ci.upper   Std.lv
Std.all
  CUDQ =~
    CUDQ_01           1.000                               1.000    1.000    0.241
0.274
    CUDQ_02           1.609    0.398    4.047    0.000    0.830    2.388    0.388
0.373
    CUDQ_03           1.672    0.389    4.294    0.000    0.909    2.435    0.404
0.425
```

```
    CUDQ_04           2.002    0.423    4.731    0.000    1.173    2.831    0.483
0.623
    CUDQ_05           1.138    0.250    4.544    0.000    0.647    1.629    0.275
0.501
    CUDQ_06           1.551    0.356    4.356    0.000    0.853    2.249    0.374
0.463
    CUDQ_07           2.048    0.431    4.746    0.000    1.202    2.893    0.494
0.676
    CUDQ_08           1.856    0.404    4.593    0.000    1.064    2.648    0.448
0.594
    CUDQ_09           2.581    0.545    4.739    0.000    1.514    3.649    0.623
0.691
    CUDQ_10           1.485    0.335    4.428    0.000    0.828    2.142    0.359
0.465
  PHQ =~
    PHQ_01            1.000                               1.000    1.000    0.655
0.618
    PHQ_02            1.100    0.099   11.129    0.000    0.906    1.293    0.721
0.696
    PHQ_03            0.998    0.097   10.267    0.000    0.808    1.189    0.654
0.638
    PHQ_04            1.006    0.097   10.395    0.000    0.816    1.195    0.659
0.659
    PHQ_05            0.851    0.097    8.763    0.000    0.660    1.041    0.557
0.537
    PHQ_06            1.187    0.108   10.981    0.000    0.975    1.398    0.777
0.696
    PHQ_07            1.130    0.103   10.926    0.000    0.928    1.333    0.741
0.695
    PHQ_08            1.011    0.097   10.395    0.000    0.821    1.202    0.663
0.665
    PHQ_09            0.877    0.097    9.073    0.000    0.688    1.067    0.575
0.558
  MOS =~
    MOSS_01           1.000                               1.000    1.000    0.472
0.307
    MOSS_03           1.847    0.355    5.208    0.000    1.152    2.542    0.872
0.555
    MOSS_04          -0.088    0.184   -0.481    0.631   -0.449    0.272   -0.042
-0.027
    MOSS_05           2.228    0.427    5.221    0.000    1.392    3.065    1.052
0.627
    MOSS_06           1.522    0.310    4.902    0.000    0.913    2.130    0.718
0.493
    MOSS_07           2.529    0.466    5.430    0.000    1.616    3.442    1.194
0.721
    MOSS_08           2.418    0.443    5.462    0.000    1.550    3.285    1.141
0.703
    MOSS_09           2.066    0.398    5.194    0.000    1.286    2.845    0.975
0.598
    MOSS_10           1.922    0.388    4.960    0.000    1.163    2.682    0.908
0.506
    MOSS_11           1.554    0.322    4.831    0.000    0.924    2.185    0.734
0.447
    MOSS_12          -0.106    0.173   -0.611    0.541   -0.446    0.234   -0.050
-0.035
```

```
Regressions:
                   Estimate  Std.Err  z-value  P(>|z|) ci.lower ci.upper   Std.lv
Std.all
  PHQ ~
    CUDQ      (b)     0.929    0.248    3.748    0.000    0.443    1.414    0.342
0.342
  MOS ~
    CUDQ      (c)     0.585    0.195    2.999    0.003    0.203    0.967    0.299
0.299
    PHQ       (e)     0.224    0.060    3.717    0.000    0.106    0.342    0.311
0.311

Intercepts:
                   Estimate  Std.Err  z-value  P(>|z|) ci.lower ci.upper   Std.lv
Std.all
   .CUDQ_01           0.614    0.045   13.640    0.000    0.525    0.702    0.614
0.697
   .CUDQ_02           1.238    0.053   23.257    0.000    1.133    1.342    1.238
1.188
   .CUDQ_03           0.854    0.049   17.584    0.000    0.759    0.949    0.854
0.899
   .CUDQ_04           0.436    0.040   11.007    0.000    0.358    0.514    0.436
0.562
   .CUDQ_05           0.209    0.028    7.451    0.000    0.154    0.264    0.209
0.381
   .CUDQ_06           0.590    0.041   14.275    0.000    0.509    0.671    0.590
0.729
   .CUDQ_07           0.410    0.037   10.975    0.000    0.337    0.483    0.410
0.561
   .CUDQ_08           0.517    0.039   13.421    0.000    0.441    0.592    0.517
0.686
   .CUDQ_09           0.728    0.046   15.801    0.000    0.638    0.819    0.728
0.807
   .CUDQ_10           0.465    0.039   11.807    0.000    0.388    0.542    0.465
0.603
   .PHQ_01            2.214    0.054   40.873    0.000    2.108    2.320    2.214
2.089
   .PHQ_02            2.172    0.053   41.058    0.000    2.069    2.276    2.172
2.098
   .PHQ_03            2.204    0.052   42.089    0.000    2.101    2.306    2.204
2.151
   .PHQ_04            2.175    0.051   42.549    0.000    2.075    2.275    2.175
2.174
   .PHQ_05            2.089    0.053   39.366    0.000    1.985    2.193    2.089
2.012
   .PHQ_06            2.175    0.057   38.129    0.000    2.063    2.287    2.175
1.948
   .PHQ_07            2.214    0.054   40.685    0.000    2.107    2.321    2.214
2.079
   .PHQ_08            1.896    0.051   37.252    0.000    1.796    1.995    1.896
1.903
   .PHQ_09            1.794    0.053   34.066    0.000    1.691    1.897    1.794
1.741
   .MOSS_01           1.569    0.078   19.990    0.000    1.415    1.723    1.569
1.021
```

```
   .MOSS_03           2.548    0.080   31.752    0.000    2.391    2.706    2.548
1.622
   .MOSS_04           2.178    0.078   27.925    0.000    2.025    2.330    2.178
1.427
   .MOSS_05           1.770    0.086   20.652    0.000    1.602    1.938    1.770
1.055
   .MOSS_06           2.726    0.075   36.587    0.000    2.580    2.872    2.726
1.870
   .MOSS_07           2.366    0.085   27.975    0.000    2.200    2.531    2.366
1.429
   .MOSS_08           1.883    0.083   22.690    0.000    1.720    2.045    1.883
1.159
   .MOSS_09           2.420    0.083   29.035    0.000    2.257    2.584    2.420
1.484
   .MOSS_10           1.431    0.092   15.613    0.000    1.251    1.610    1.431
0.798
   .MOSS_11           2.360    0.084   28.164    0.000    2.196    2.525    2.360
1.439
   .MOSS_12           2.016    0.073   27.468    0.000    1.872    2.159    2.016
1.404

Variances:
                   Estimate  Std.Err  z-value  P(>|z|) ci.lower ci.upper   Std.lv
Std.all
   .CUDQ_01           0.717    0.053   13.576    0.000    0.613    0.820    0.717
0.925
   .CUDQ_02           0.934    0.070   13.259    0.000    0.796    1.072    0.934
0.861
   .CUDQ_03           0.740    0.056   13.123    0.000    0.630    0.851    0.740
0.820
   .CUDQ_04           0.367    0.031   11.757    0.000    0.306    0.429    0.367
0.611
   .CUDQ_05           0.226    0.018   12.699    0.000    0.191    0.260    0.226
0.749
   .CUDQ_06           0.514    0.040   12.924    0.000    0.436    0.592    0.514
0.786
   .CUDQ_07           0.290    0.026   11.125    0.000    0.239    0.341    0.290
0.543
   .CUDQ_08           0.367    0.030   12.108    0.000    0.308    0.427    0.367
0.647
   .CUDQ_09           0.426    0.040   10.766    0.000    0.348    0.503    0.426
0.523
   .CUDQ_10           0.465    0.036   12.938    0.000    0.394    0.535    0.465
0.783
   .PHQ_01            0.695    0.056   12.505    0.000    0.586    0.803    0.695
0.618
   .PHQ_02            0.553    0.047   11.791    0.000    0.461    0.645    0.553
0.516
   .PHQ_03            0.622    0.051   12.272    0.000    0.523    0.721    0.622
0.592
   .PHQ_04            0.567    0.047   12.151    0.000    0.475    0.658    0.567
0.566
   .PHQ_05            0.768    0.059   12.954    0.000    0.652    0.884    0.768
0.712
   .PHQ_06            0.642    0.055   11.736    0.000    0.535    0.749    0.642
0.515
```

```
   .PHQ_07            0.586    0.050   11.717    0.000    0.488    0.684    0.586
0.516
   .PHQ_08            0.553    0.046   12.076    0.000    0.463    0.642    0.553
0.557
   .PHQ_09            0.731    0.057   12.845    0.000    0.620    0.843    0.731
0.689
   .MOSS_01           2.137    0.158   13.533    0.000    1.828    2.447    2.137
0.906
   .MOSS_03           1.707    0.136   12.542    0.000    1.440    1.974    1.707
0.692
   .MOSS_04           2.327    0.168   13.836    0.000    1.997    2.657    2.327
0.999
   .MOSS_05           1.707    0.144   11.844    0.000    1.425    1.990    1.707
0.607
   .MOSS_06           1.610    0.125   12.843    0.000    1.364    1.855    1.610
0.757
   .MOSS_07           1.313    0.123   10.687    0.000    1.072    1.554    1.313
0.479
   .MOSS_08           1.333    0.121   11.012    0.000    1.096    1.571    1.333
0.506
   .MOSS_09           1.710    0.141   12.120    0.000    1.434    1.987    1.710
0.643
   .MOSS_10           2.393    0.188   12.715    0.000    2.024    2.762    2.393
0.744
   .MOSS_11           2.151    0.164   13.079    0.000    1.829    2.474    2.151
0.800
   .MOSS_12           2.060    0.149   13.834    0.000    1.768    2.352    2.060
0.999
    CUDQ              0.058    0.024    2.466    0.014    0.012    0.105    1.000
1.000
   .PHQ               0.379    0.061    6.199    0.000    0.259    0.499    0.883
0.883
   .MOS               0.167    0.060    2.788    0.005    0.050    0.285    0.751
0.751

R-Square:
                   Estimate
    CUDQ_01           0.075
    CUDQ_02           0.139
    CUDQ_03           0.180
    CUDQ_04           0.389
    CUDQ_05           0.251
    CUDQ_06           0.214
    CUDQ_07           0.457
    CUDQ_08           0.353
    CUDQ_09           0.477
    CUDQ_10           0.217
    PHQ_01            0.382
    PHQ_02            0.484
    PHQ_03            0.408
    PHQ_04            0.434
    PHQ_05            0.288
    PHQ_06            0.485
    PHQ_07            0.484
    PHQ_08            0.443
    PHQ_09            0.311
```

```
    MOSS_01          0.094
    MOSS_03          0.308
    MOSS_04          0.001
    MOSS_05          0.393
    MOSS_06          0.243
    MOSS_07          0.521
    MOSS_08          0.494
    MOSS_09          0.357
    MOSS_10          0.256
    MOSS_11          0.200
    MOSS_12          0.001
    PHQ              0.117
    MOS              0.249

Defined Parameters:
                   Estimate  Std.Err  z-value  P(>|z|) ci.lower ci.upper   Std.lv
Std.all
    in_CUDQ_PH_MOS    0.208    0.075    2.758    0.006    0.060    0.356    0.106
0.106
    total_CUDQ_MOS    0.793    0.238    3.335    0.001    0.327    1.258    0.405
0.405


>
> # Remaining extraction values
>
> parameterEstimates(fit_redesign, standardized = TRUE) %>%
+   filter(op %in% c("~", ":="))
                   lhs op     rhs                label   est    se     z pvalue ci.
lower ci.upper std.lv std.all
1                  PHQ  ~    CUDQ                    b 0.929 0.248 3.748  0.000
0.443    1.414  0.342   0.342
2                  MOS  ~    CUDQ                    c 0.585 0.195 2.999  0.003
0.203    0.967  0.299   0.299
3                  MOS  ~     PHQ                    e 0.224 0.060 3.717  0.000
0.106    0.342  0.311   0.311
4 indirect_CUDQ_PH_MOS :=     b*e indirect_CUDQ_PH_MOS 0.208 0.075 2.758  0.006
0.060    0.356  0.106   0.106
5       total_CUDQ_MOS := c+(b*e)       total_CUDQ_MOS 0.793 0.238 3.335  0.001
0.327    1.258  0.405   0.405
```
